# Crystal Structure and Catalytic Mechanism of CouO, a Versatile C-Methyltransferase from *Streptomyces rishiriensis*

**DOI:** 10.1371/journal.pone.0171056

**Published:** 2017-02-02

**Authors:** Tea Pavkov-Keller, Kerstin Steiner, Mario Faber, Martin Tengg, Helmut Schwab, Mandana Gruber-Khadjawi, Karl Gruber

**Affiliations:** 1 Institute of Molecular Biosciences, University of Graz, Graz, Austria; 2 ACIB, Austrian Centre of Industrial Biotechnology GmbH, Graz, Austria; 3 Institute of Molecular Biotechnology Graz University of Technology, Graz, Austria; 4 Institute of Organic Chemistry, Graz University of Technology, Graz, Austria; Universität Stuttgart, GERMANY

## Abstract

Friedel–Crafts alkylation of aromatic systems is a classic reaction in organic chemistry, for which regiospecific mono-alkylation, however, is generally difficult to achieve. In nature, methyltransferases catalyze the addition of methyl groups to a wide range of biomolecules thereby modulating the physico-chemical properties of these compounds. Specifically, S-adenosyl-L-methionine dependent *C*-methyltransferases possess a high potential to serve as biocatalysts in environmentally benign organic syntheses. Here, we report on the high resolution crystal structure of CouO, a *C*-methyltransferase from *Streptomyces rishiriensis* involved in the biosynthesis of the antibiotic coumermycin A1. Through molecular docking calculations, site-directed mutagenesis and the comparison with homologous enzymes we identified His120 and Arg121 as key functional residues for the enzymatic activity of this group of *C*-methyltransferases. The elucidation of the atomic structure and the insight into the catalytic mechanism provide the basis for the (semi)-rational engineering of the enzyme in order to increase the substrate scope as well as to facilitate the acceptance of SAM-analogues as alternative cofactors.

## Introduction

Methylation is one of the most essential reactions in all living organisms and plays an important role in the expression, structure, and function of biological molecules such as proteins, DNA/RNA, and small molecules. The methyl groups are selectively introduced by methyltransferases (MTases), a large group of enzymes that can be divided into several subclasses based on their structural features. The most common class of MTases is class I, possessing a Rossmann-like fold and utilizing *S*-adenosyl-L-methionine (SAM) as a methyl donor [1]. For this group, a general S_N_2-like nucleophilic substitution mechanism for methyl transfer is proposed yielding *S*-adenosyl-L-homocysteine (SAH) and the methylated substrate. Natural-product MTases are the functionally most diverse class of MTases and methyl groups are added to S, N, O or C atoms. The proposed catalytic mechanisms include general acid-base catalysis, metal-based catalysis as well as proximity and desolvation effects not requiring catalytic amino acids [2].

**Fig 1:**
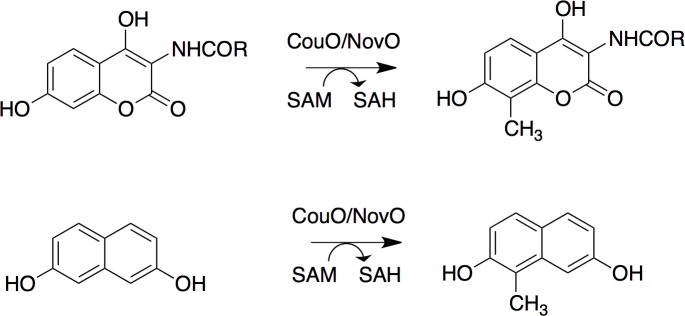
Methyltransfer catalyzed by the *C*-methyltransferases CouO and NovO. The alkylation of aromatic substrates with coumarin and naphthalene scaffold is shown. The enzymes also accept SAM-analogues to yield allyl-, propargyl- and benzyl-arenes [3].

Previously, we reported on the Friedel–Crafts alkylation catalyzed by SAM-dependent *C*-methyltransferases, CouO from *Streptomyces rishiriensis* and NovO from *Streptomyces spheroids* [3–5]. In nature, CouO and NovO catalyze one of the final steps in the biosyntheses of the antibiotics coumermycin A_1_ and novobiocin, *i*.*e*. the methylation of the C-8 atom of the coumarin moiety (Fig 1) [3, 6]. Remarkably, both enzymes accept a broad range of substrates as well as chemically modified cofactors as alkyl donors [3]. Using CouO and NovO allyl-, propargyl- and benzyl-arenes of different coumarin scaffold derivatives could be produced in moderate to high yields with excellent regioselectivity. In addition, both enzymes showed activity towards various naphthalene derivatives as well [3].

Despite extensive studies on *C*-methyltransferases, the chemical mechanism is still poorly understood and it is not completely clear which amino acid residues are involved in substrate binding and catalysis. Recently, a crystal structure of the *C*-methyltransferase Coq5 was reported [7]. Coq5 catalyzes the methylation step in coenzyme Q biosynthesis pathway, the conversion of 2-methoxy-6-polyprenyl-1,4-benzoquinone to 2-methoxy-5-methyl-6-polyprenyl-1,4-benzoquinone. Based on this structure, a catalytic mechanism was proposed involving Arg-201 as a general base that initiates catalysis with the help of a water molecule [7].

Here, we present the high-resolution crystal structure of the *C*-methyltransferase CouO from *Streptomyces rishiriensis* in complex with SAH. Based on this structure and in combination with molecular docking studies and site-directed mutagenesis we identified key functional residues for enzymatic activity and propose an alternative catalytic mechanism for this group of enzymes. These new insights will subsequently enable the (semi)-rational design of CouO and NovO variants for the alkylation of target substrates, which serve as intermediates for various follow-up reactions and applications.

## Materials and Methods

### General

All commercially available reagents and solvents were used without further purification unless otherwise stated. Demineralized water for HPLC was filtrated through a 0.2 μm cellulose nitrate membrane filter prior to use. The substrate for the activity assay, *N*-(4,7-dihydroxy-2-oxo-2*H*-chromen-3-yl)benzamide, was synthesized as described previously [3, 5]. Materials for molecular biology were obtained from Thermo Fisher Scientific (Waltham, MA, USA), if not specifically stated otherwise.

### Structure determination of CouO

X-ray diffraction data were collected at DESY, Hamburg to 2.05 Å resolution on a crystal belonging to the monoclinic space group *P*2_1_ with unit cell dimensions a = 33.1 Å, b = 82.9 Å, c = 76.9 Å and β = 96.9°, respectively [8]. The structure was solved by a combination of molecular replacement, extensive manual rebuilding and the application of the phenix.mr_rosetta protocol [9]. By limiting the search template to a truncated common core-structure of *S*-adenosyl-l-methionine (SAM)-dependent methyltransferases an initial solution with two molecules in the asymmetric unit was obtained using the program Phaser [10]. As less than 40% of total residues were used for molecular replacement, only a part of the structure was visible in the electron density map. Manual (re)building was performed in Coot [11] and the obtained partial model was used as starting model for the phenix.mr_rosetta [9] protocol as available in Phenix [12]. The resulting electron density map was used for manual rebuilding and stepwise addition of missing residues interspersed with refinement cycles in Refmac5 [13], until the electron density for the rest of the molecule appeared. The model was completed using ARP/wARP [14] as included in CCP4 [15]. The manual rebuilding and refinement was continued using Coot and Refmac5. A randomly chosen set of 5% of the reflections was not used in the refinement, but was set aside for R_free_ calculations [16]. The stereochemistry and geometry were analyzed using Molprobity [17] and the agreement between the atomic model and X-ray data was checked with SFCHECK [18]. In the Ramachandran plot, all residues of the model are located in the most favorable or allowed regions. Detailed statistics are shown in Table 1. The atomic coordinates and structure factors have been deposited in the Protein Data Bank as entry 5M58.

**Table 1:** CouO structure refinement and validation statistics.

**Data collection**	
Wavelength (Å)	1.0507
Space group	*P*2_1_
Unit cell parameters (Å, °)	a = 33.07, b = 82.95, c = 76.88, β = 96.93
Resolution (Å)	41.5–2.05 (2.16–2.05)
*R*_*merge*_	0.138 (0.532)
Completeness (%)	97.9 (94.2)
<I/σ(I)>	6.0 (2.5)
Multiplicity	3.5 (3.0)
**Refinement**	
*R*_work_ / *R*_free_	0.208 / 0.264
No. of protein atoms	3668
No. of water molecules	293
Mean B factor	22.35
R.m.s. deviations	
Bond lengths (Å)	0.010
Bond angles (°)	1.442
Ramachandran outliers (%)	0
Most favored residues (%)	97.4
**PDB**	5M58

### Structure analysis and docking calculations

Sequences were aligned using the program T-coffee [19] and the alignment was graphically rendered using ESPript 3.0 [20]. The Dali server [21] and the PDBeFold service at European Bioinformatics Institute (http://www.ebi.ac.uk/msd-srv/ssm) [22] were used to identify similar protein structures in the PDB. Protein interfaces were analyzed with the PDBePISA webserver (http://www.ebi.ac.uk/pdbe/prot_int/pistart.html) [23]. Structures were superimposed using the program SSM Superposition [22] as implemented in the program Coot. All structure-related pictures were generated using PyMOL (http://www.pymol.org). Cavity analyses were performed using the LIGSITE algorithm [24] as implemented in the CaSoX plugin for Pymol. For the analysis of the hydrophobicity of the cavities the hydrophobic calculation module of the program VASCo [25] was used.

Docking calculations were performed using the program VINA [26] employing default parameters. Partial charges were assigned according to the AMBER03 force field [27]. The setup was performed within the YASARA molecular modeling program [28] using both SAH and SAM present in the active site as well as with protonated and unprotonated forms of the substrates *N*-(4,7-dihydroxy-2-oxo-2*H*-chromen-3-yl)benzamide, 3-amino-4,7-dihydroxycoumarin and 4,7-dihydroxycoumarin. For each ligand 25 docking runs were performed without water molecules present. During energy minimization in YASARA water molecules were added automatically.

### Cloning and mutagenesis

The mutations were introduced by overlap-extension PCR. The construct pET26b-CouO [3] was used as template for two separate PCR reactions, one containing the outer primer CouO(pMS)_for and a reverse mutagenic primer and the other containing the outer primer CouO(pMS)_rev and a forward mutagenic primer (S1 Table). The PCR reaction was performed in a total volume of 50 μL containing the supplied reaction buffer, 1 ng of template DNA, 0.2 μM of each primer, 0.2 mM of each dNTP, and 1 U Phusion Polymerase. The PCR program was as follows: 1 min at 98°C, 30 cycles of denaturation at 98°C for 20 s, annealing at 55°C for 30 s and extension at 72°C for 1 min, and a final extension step at 72°C for 7 min. The PCR products were purified after separation from a 1% agarose gel (Wizard SV PCR and Clean-Up System; Promega, Madison, WI, USA). The two PCR products were combined by a second PCR containing both outer primers. The purified PCR products were used for Gibson cloning [29] into the pMS470Δ8 vector [30], which was linearized with *Nde*I/*Hind*III. The mixture was transformed into *E*. *coli* TOP10F‘ cells (Life Technologies, Carlsbad, CA, USA). All constructs were confirmed by sequencing (Microsynth AG, Balgach, Switzerland).

### Protein expression

*E*. *coli* TOP10F‘ cells harboring the different plasmids were grown over night at 37°C in 2xTY (50 mL in 100 mL shaking flask) medium supplemented with 100 mg/L ampicillin (pMS470-constructs). They were used for the inoculation of the main cultures (400 mL 2xTY media in 1 L baffled flasks) to an OD600 of 0.05. The cells were grown to an OD_600_ of ~0.8 at 37°C and 130 rpm. Protein expression was induced by the addition of 0.1 mM isopropyl-β-D-thiogalactoside (IPTG) and cultivation was continued at 25°C for 20 h and 130 rpm. The cultures were harvested by centrifugation at 3.600 g for 15 minutes at 4°C. The cell pellets were stored at -20°C until further use, when they were resuspended in 25 mL of 50 mM sodium phosphate pH 7.5 and disrupted by sonication (Branson sonifier S-250; 80% duty cycle, output control 7) for 6 minutes under continuous cooling. Cell free lysates were obtained by centrifugation (48.000 g, 1 h, 4°C). The protein concentrations of the lysates were determined with the Bio-Rad Protein Assay (Bio-Rad, Hercules, CA, USA). For gel electrophoresis, NuPAGE^®^ 4–12% Bis-Tris Gels, 1.0 mm, (Life Technologies, Carlsbad, CA, USA) were used with NuPAGE MES SDS Running Buffer. The percentage of expressed protein in cleared lysates was determined by quantifying the respective band on a Coomassie-stained SDS-PA gel using a G-box HR16 device (Syngene, Synoptics, Cambridge, UK).

The variants CouO-H15A, -H15N, -R24A and -R121L showed the same expression level as soluble protein as CouO-WT. The variants CouO-H117A and -Y216F were slightly less expressed as soluble protein, whereas the R121A variant was slightly better expressed. In the soluble fractions of CouO-H117S and -H120N clearly less target protein was expressed and the band of H120A was hardly detectable in the soluble fraction (S7 Fig and S2 Table).

### Activity assay

The activity assay was carried out according to Tengg *et al*. [4]. Soluble protein fractions of recombinant *E*. *coli* cells expressing CouO and its variants were incubated in 0.1 ml scale in a thermomixer at 30°C and 1000 rpm for 24 h. The reactions contained 0.5 mM substrate *N*-(4,7-dihydroxy-2-oxo-2*H*-chromen-3-yl)benzamide (10 mM stock solution prepared in DMSO), 2 mM SAM- dihydrochloride (Sigma–Aldrich, St. Louis, USA) in 50 mM sodium phosphate buffer pH 7 and 0.1 mg/ml BSA (in 50 mM sodium phosphate buffer of pH 7) (S6 Fig). The soluble enzyme content in the assay was calculated to match H120A, the variant with the least soluble protein. Therefore, a calculated amount of 28.6 μg Mtase was used for the assays (S2 Table). Following the incubation at 30°C the reactions were stopped by heating at 80°C for 15 min and the denatured protein was removed via centrifugation at 16,000 g for 15 min. The supernatant was filtrated using centrifugal filter with a modified polyethersulfone (PES) membrane with a cutoff of 3 kDa (VWR, Radnor, USA). 5 μL of the resulting clear aqueous solutions were analyzed by HPLC (see Supporting Information). All reactions were carried out in triplicate. Using the empty vector pMS470d8 no CouO activity was observed, therefore all blank reactions were carried out only with the 50 mM sodium phosphate buffer pH 7.

### HPLC-MS

Samples from the activity assays were analyzed using a Shimadzu Nexera HPLC-MS system comprising two Nexera LC-30AD pump modules, Nexera SIL-30AC auto sampler, CTO-20AC prominence column oven, SPD-M20A prominence diode array detector, CBM-20A prominence communications bus module, FCV-20AH2 valve unit and LCMS-2020 quadrupole mass detector. The analyses were carried out on an Agilent Poroshell 120 EC-C18, 100x3 mm, 2.7 μm column using a solvent gradient (see Figures A-L in S1 File).

## Results and Discussion

The crystal structure of CouO was determined by molecular replacement using the truncated common core-structure obtained by superposition of available methyltransferase structures with similar sequences (maximum identity <30%), which proved to be a challenging task. An important step was truncating the common core-structure to only those parts that were really conserved and obtaining the correct initial solution with two common core-structures in the asymmetric unit. Less than 40% of the total residues were thus used for initial phasing. Still, we continued with the manual rebuilding and refinement by adding only a few extra residues (max. 4) per refinement cycle until the electron density for the whole molecule appeared. After extensive rebuilding and refinement, two CouO molecules in the asymmetric unit could be completely modeled into the residual electron density. In addition, one SAH cofactor molecule was present in each chain (S1 Fig). Detailed statistics of the structure determination and refinement are listed in Table 1.

**Fig 2:**
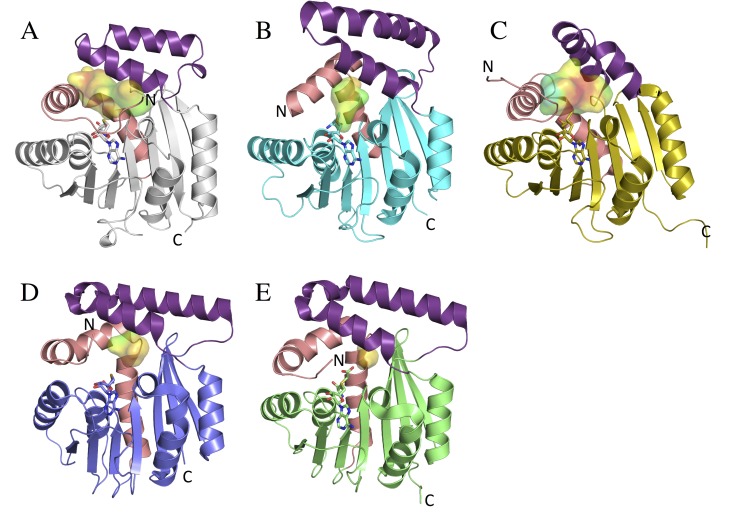
Overall structures of CouO and similar methyltransferases. Monomers of A) CouO, B) Coq5 (PDB: 4obw), C) mmp1179 (PDB: 3dlc), D) YexO (PDB: 1im8) and CmoA (PDB: 4gek) are shown in ribbon representations. The cofactors (SAH in CouO, SAM in Coq5 and mmp1179, Cx-SAM in CmoA and SAI in YecO) are shown in sticks representations and the active site cavities are shown as surfaces. The cavity surfaces are colored according to their hydrophobicity (red-hydrophobic to blue-hydrophilic). The main differences are observed in the conformation of the cap-domain (violet) and in the position of the N-terminal part consisting of a loop and the first α-helix (salmon).

The overall CouO structure exhibits a core with a Rossmann-like α/β-fold typical of class I SAM-dependent methyltransferases and a cap-domain accommodating two α-helices (Fig 2A). An analysis of protein-protein interfaces between the two CouO chains in the asymmetric unit using the PDBePISA server identified a dimer interface that is formed by the α-helices of the cap-domain and mainly involves hydrophobic interactions (S2 Fig). Gel-filtration chromatography, performed as a last step of the protein purification, also indicated that CouO exists as a dimer in solution (S2 Fig). Comparing the final structure of CouO with structures available in the PDB, the structures of the *C*-methyltransferase Coq5 (PDB: 4obw, 4obx) [7], the putative SAM-dependent methyltransferase (mmp1179) from *Methanococcus maripaludis* (PDB: 3dlc, Joint Center for Structural Genomics), CmoA from *E*.*coli* (PDB: 4gek) [31] and YecO from *Haemophilus influenza* (PDB: 1im8) [32] were identified as the closest matches with a similar two-helix architecture of the cap-domain. Similar to CouO, Coq5 and CmoA were also reported to form dimers [7, 31]. CmoA and YecO are involved in the biosynthesis of 5-oxyacetyl uridine in Gram-negative bacteria catalyzing the formation of carboxy-*S*-adenosyl-L-methionine (Cx-SAM) from SAM and prephenate [31].

These structurally very similar enzymes mainly differ in the conformation of the cap-domain and the position of the N-terminal part consisting of a loop and a first α-helix (Fig 2). The cofactor-binding site is situated between these two regions and accommodates different forms of cofactors: SAH, SAM, SAI (*S*-adenosyl-L-homoselenocysteine) and Cx-SAM. For Rossmann-fold enzymes that use different cofactors, a canonical motif defined by a carboxylate side chain at the tip of the second β-strand (β2-Asp/Glu) with a unique geometry has been identified [33]. In CouO, this motif is also present and involves Asp70 forming bidentate interactions with both hydroxyl groups of the ribose. The N-terminal tail of the protein, which is not part of the canonical Rossmann-fold, wraps around the bound cofactor and the side chain of Glu4 forms hydrogen bonds with the amino group of the adenine moiety of SAM/SAH (S3 Fig). In this conformation of the N-terminal loop the cofactor would not be able to enter the cavity, indicating and necessitating an increased flexibility of this region upon binding/release of the cofactor. Comparing the SAM conformation(s) as reported for Coq5 we observe some differences to the conformation of SAH in CouO or SAM in mmp1179. It should be mentioned, however, that Coq5 was crystallized as a truncated version comprising residues Ser61 to Val307, therefore missing the potentially important N-terminal part.

**Fig 3:**
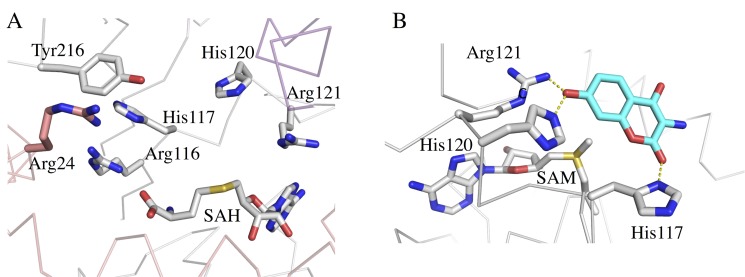
Active site of CouO. A) Amino acid residues and SAH cofactor in the active site of CouO. B) The lowest energy docking mode (as calculated using the program YASARA) places the coumarin moiety (in cyan) in the vicinity of the SAM cofactor. Amino acid residues His120 and Arg121 are situated about 3 Å from the hydroxyl-oxygen at C-7 of the substrate. These interactions are indicated as yellow dashed lines.

Structure examination of CouO located a cavity in the vicinity of the SAH binding site (Fig 2A). This cavity is lined by the amino acids Ile6, Glu10, Phe14, Met17, Tyr25, Arg116, His117, His120, Arg121, Phe147, Phe164, Trp170, Met174, Trp178 and Tyr216. Hydrophobic residues are arranged along the central part of the cavity, directly above the sulfur atom of SAH, whereas His and Arg residues are situated on the periphery (Fig 3A). Cavities are also present at the respective locations in the four other methyltransferases, with differences in their size and hydrophobic properties (Fig 2). In Coq5, CmoA and YecO the N-terminal α-helix closes the cavity from one side thus significantly decreasing its size. In CouO and mmp1179, on the other hand, the position of this N-terminal α-helix allows for more space resulting in a much bigger cavity and more space for binding larger substrates.

**Fig 4:**
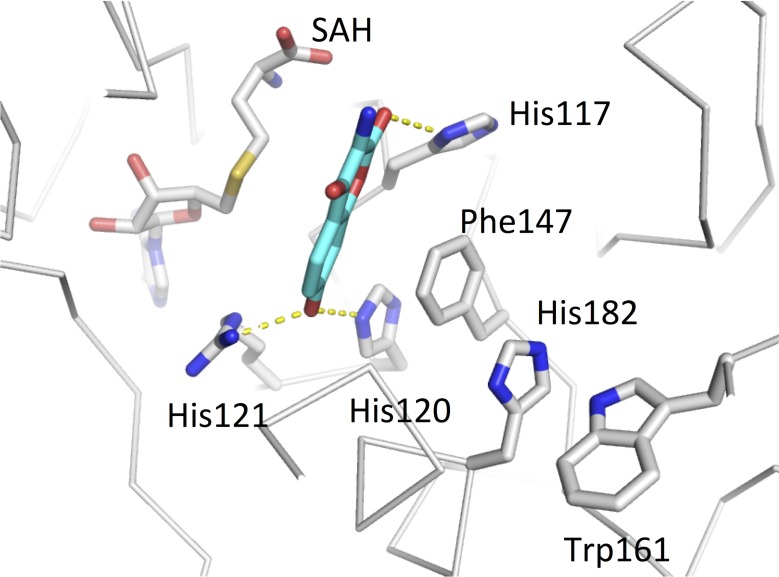
π-stacking interactions between side chains in the active site. The interaction between His120 and Phe147 very likely ensures the optimal orientation of the imidazole group of the histidine for the deprotonation of the substrate hydroxyl group. The coumarin moiety (placed by molecular docking calculations) is show in cyan. Hydrogen bonding interactions are indicated by dashed yellow lines.

Docking calculations with the CouO structure using several ligands containing a coumarin scaffold identified His120 and Arg121 as most likely crucial for the enzymatic activity. The lowest energy docking mode places the coumarin moiety in the vicinity of the cofactor, whereas His120 and Arg121 are situated about 3 Å from the hydroxyl-oxygen at C-7 of the substrate (Fig 3B). In this binding mode C-8 would be in an appropriate position for the nucleophilic attack on the methyl group of SAM, *i*.*e*. in line with the methyl group and the sulfur atom of the cofactor, which is required by the classic S_N_2 reaction mechanism proposed for most other MTases [1]. Furthermore, π-stacking interactions between the side chains of His120 and Phe147 very likely ensure the optimal orientation of the imidazole group of the histidine and may contribute to the activation of this residue for the deprotonation of the substrate hydroxyl group (Fig 4). The residues His117 and Arg116 could be additionally involved in the correct positioning of the substrate moiety for the methyl-transfer reaction to take place.

**Fig 5:**
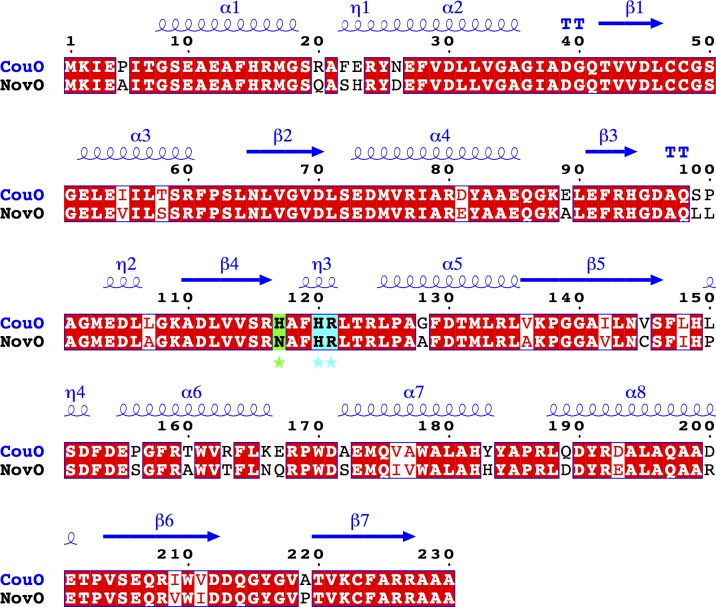
Sequence comparison of NovO and CouO. Identical residues are shown in red boxes. Secondary structure elements of CouO are shown in blue above the sequence alignment. Important amino acid residues in the active site are marked with an asterisk and shown in cyan (identical) and green (similar) boxes. This figure was prepared using ESPript 3.0 [20].

A homology model of NovO was generated using the CouO structure as a template (85% sequence identity, 90% similarity). Both enzymes accept substrates with identical architecture and vary only in respective conversion rates [3]. Most of the residues situated in the active site are highly conserved in both enzymes (Fig 5), suggesting a common mechanism. The only differences are exchanges of His117 and Val145 in CouO to Asn117 and Cys145 in NovO (S4 Fig).

**Fig 6:**
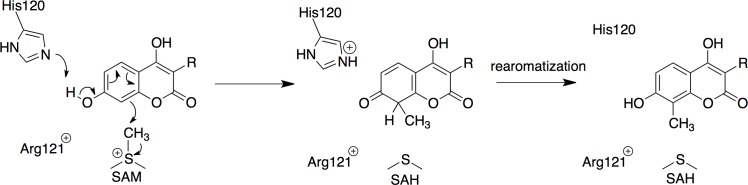
Proposed reaction mechanism. Reaction mechanism for the Friedel–Crafts alkylation catalyzed by SAM-dependent *C*-methyltransferases, CouO from *Streptomyces rishiriensis* and NovO from *Streptomyces spheroides*.

Based on the crystal structure of CouO, the docking studies and the comparison with NovO, we propose a catalytic mechanism for both enzymes (Fig 6). The methylation most likely proceeds *via* base-assisted deprotonation of the hydroxyl group followed by a nucleophilic attack of the newly generated resonance stabilized phenolate-ion at C-7 of the substrate on the reactive methyl group of SAM. With His120 playing the role of a general base, the positively charged Arg121 would stabilize the negatively charged intermediate. The C-8 carbon of the coumarin scaffold attacks the methyl group of SAM generating a methylated, non-aromatic intermediate and SAH. Subsequent tautomerization leads to the (re)formation of the aromatic system. A superposition of the structures of CouO and Coq5 places the proposed general bases (His120 in CouO and Arg201 in Coq5) in very similar positions relative to the cofactor (S5 Fig). In the case of Coq5 [7], however, the deprotonation of the substrate involves a water molecule as mediator, which is activated by the arginine residue, rather than a direct interaction between the base and the substrate.

To validate the proposed mechanism and to study the effect of individual residues on the catalytic activity of the enzyme, the amino acid residues Arg24, His117, His120, Arg121 and Tyr216 were chosen as targets for protein engineering by site-directed mutagenesis. In case of NovO, the exchange of His15 by glutamine, asparagine, lysine or arginine also had some impact on the enzymatic activity [4]. Although this residue is not located in the active site but in an adjacent α-helix, we still included it in our mutagenesis studies because of the close similarity of the two enzymes.

**Fig 7:**
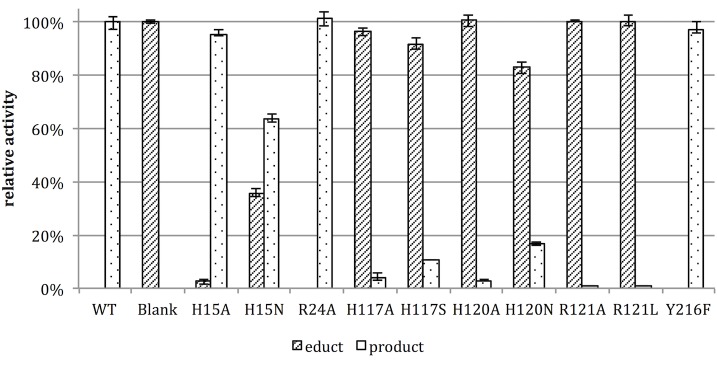
Relative activities of the CouO variants. The individual activities of the variants in the methylation of the coumarin compound *N*-(4,7-dihydroxy-2-oxo-2*H*-chromen-3-yl)benzamide (educt) are expressed as % product formation relative to the wild-type enzyme. The reported values are mean values from triplicate measurement and the bars indicate the minimum and maximum values obtained.

The resulting activities of the CouO variants (normalized to the apparent expressed protein content, S7 Fig and S2 Table) are shown in Fig 7. In line with our proposed mechanism (Fig 6), the exchange of residues directly involved in interactions with the substrate, such as His120, Arg121 and His117, led to a significant reduction of the enzyme activity. The exchange of Arg121 resulted in an almost complete loss of the activity, whereas other tested amino acid exchanges showed little (H15A and H15N) or no (R24A and Y216F) influence on the enzyme activity. The observed residual activity of variants H120A and H120N could be explained by some fraction of the substrate being deprotonated under the reaction conditions (pH 7).

It has been shown that CouO and NovO accept modified cofactors and are able to transfer larger groups (*e*.*g*. allyl or benzyl) onto the substrate [3–5]. Interestingly, the structurally similar enzyme CmoA from *E*. *coli* also accepts Cx-SAM as its natural cofactor [31], indicating some degree of cofactor promiscuity in these enzymes. The present CouO structure indeed indicates that there already is room to accommodate larger SAM-analogs. Exchanges of active site residues that are not directly involved in the reaction mechanism–*e*.*g*. the replacement of the more bulky residues Ile6, Phe14, Trp170, Met75, Arg24 and Tyr216 with smaller counterparts–might provide additional means to widen the cofactor and substrate scope of the enzyme.

## Conclusions

Since methylation is known to enhance the bioactivity of many natural products [34, 35] CouO and NovO offer substantial promise as biocatalysts for the chemoenzymatic synthesis of novel compounds with therapeutic potential. In particular due to their excellent chemo- and regioselectivity, which favors them over chemical methylation, MTases have a great potential for biotechnological and biomedical applications [36]. The integration of the MTases in a multistep enzyme cascade that addresses the cofactor regeneration limitation already showed to be successful with other enzymes of this family [37]. The gathered information about the three-dimensional structure and the enzymatic mechanism can serve as the basis for rationally engineered CouO/NovO variants with a broadened acceptance of SAM analogues that carry extended carbon chains, as well as the consecutive alkylation of preferable substrates.

## Supporting Information

S1 Fig Fo-Fc omit density map (contoured at 3σ) of the SAH region. The SAH is shown as grey sticks.
(TIF)

S2 Fig CouO dimer. A) CouO dimer interface formed between the α-helices of the cap-domain that mainly involves hydrophobic interactions as identified by the PDBePISA server. The SAH cofactor is shown in sticks. B) The final step of CouO purification involved size exclusion chromatography (HiLoad 16/60 Superdex 200 pg column (GE Healthcare), 1 ml/min, Hepes pH 7, 100mM NaCl). Red trace: Bio-Rad gel filtration standard peaks: 1–670 kDa, thyroglobulin, 2–158 kDa, γ-globulin, 3–44k Da, ovalbumin, 4–17 kDa, myoglobin, 5–1.34 kDa, vitamin B_12_. In the blue trace, peak A corresponds to aggregates, whereas samples from peak B were used for crystallization. A least-squares fit (log(MW) *vs*. elution volume) yielded a molecular weight of 52.4 kDa for peak B.
(TIF)

S3 Fig Close-up view of residues involved in SAH-binding. Amino acid residues present in the canonical motif, Asp70, and in the N-terminal tail, Glu6, are shown in a stick representation. The coloring scheme is the same as in Fig 1. Hydrogen bonding interactions are shown as yellow dashed lines.
(TIF)

S4 Fig CouO–NovO active site comparison. A homology model of NovO was generated using the CouO structure as a template (85% sequence identity, 90% similarity). Selected amino acid residues in and around the active site are shown in a stick representation. Residues 117 and 145 differ in two enzymes. The coloring scheme for CouO is the same as in Fig 1, residues differing in NovO are shown in green.
(TIF)

S5 Fig Superposition of CouO (grey) and Coq5 (magenta). The residues suggested to be involved in catalysis, as well as the SAH/SAM cofactor are shown in sticks representations.
(TIF)

S6 Fig Activity assay. 0.5mM *N*-(4,7-dihydroxy-2-oxo-2*H*-chromen-3-yl)benzamide was used as substrate.
(TIF)

S7 Fig SDS-PAGE analysis of soluble (left) and insoluble fractions (right) obtained by sonication and centrifugation of *E*. *coli* TOP10F’ expressing CouO and variants thereof (pMS470 vector). 1: H15A, 2: H15N, 3: R24A, 4: H117A, 5: H117S, 6: H120A, 7: H120N, 8: R121A, 9: R121L, 10: Y216F, 11: WT, 12: pMS470d8. St: Page Ruler Prestained Protein Ladder (Fermentas). The arrow indicates the location of the target protein (expected MW = 26kDa).
(TIF)

S1 File HPLC (UV) chromatograms and MS analyses of the conversions catalyzed by wild type CouO and variants thereof. (PDF containing 12 figures, Figures A to L in S1 File)
(PDF)

S1 Table Sequences of primers used for cloning and mutagenesis in this study. Sequences of restriction sites are underlined, start and stop codon are indicated with boxes. Bold red letters indicate the mutated bases.
(PDF)

S2 Table Percentage of wild type enzyme and CouO variants in cell-free lysates. (PDF)
