# Supplementary figures and images for: Crystal Structure and Catalytic Mechanism of CouO, a Versatile C-Methyltransferase from Streptomyces rishiriensis
Source: PLoS One. 2017 Feb 2;12(2):e0171056. doi: 10.1371/journal.pone.0171056 (PMC5289526; doi:10.1371/journal.pone.0171056)

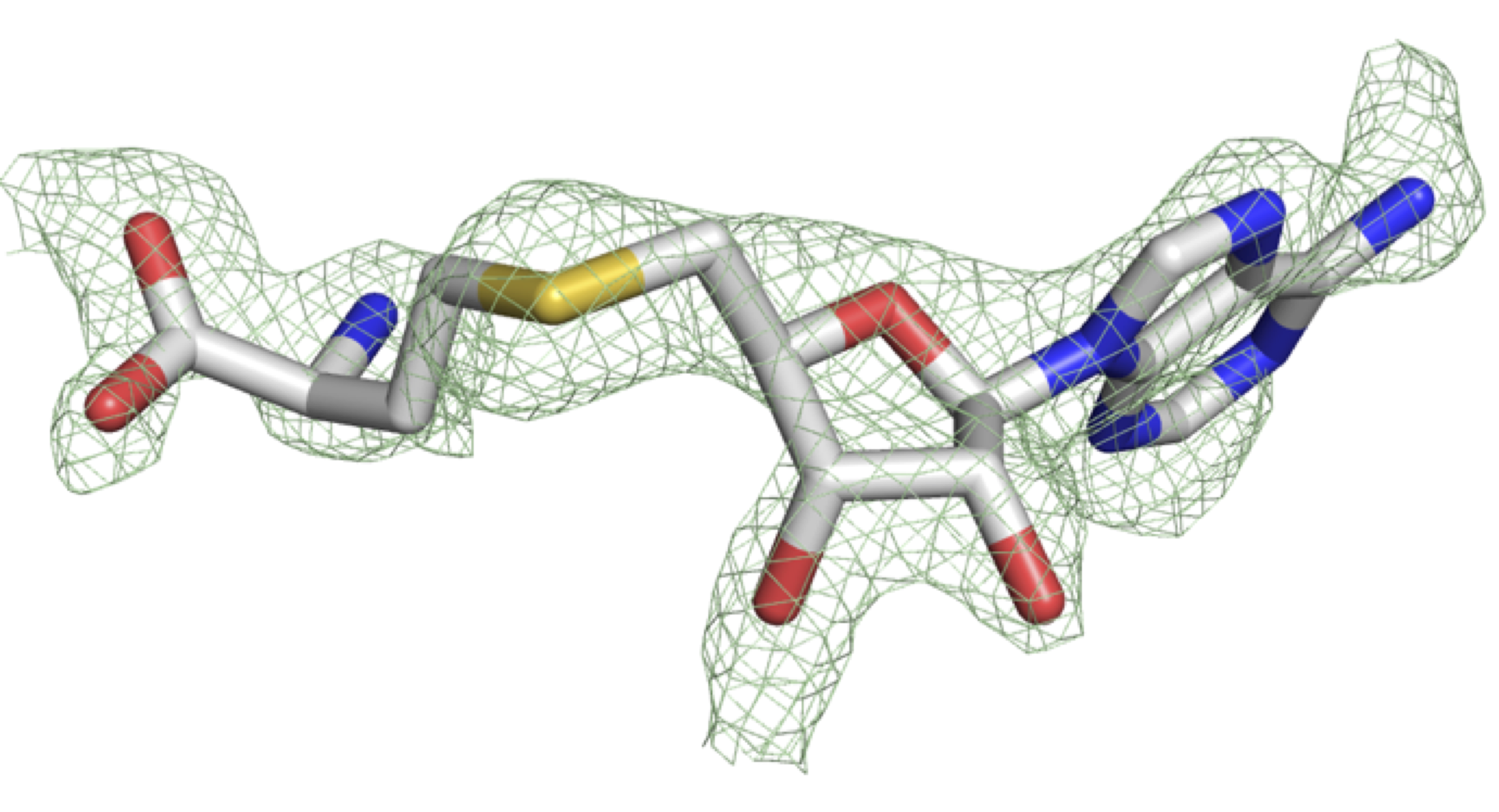

Supplement: S1 Fig — The SAH is shown as grey sticks. (TIF) [file pone.0171056.s001.tif]

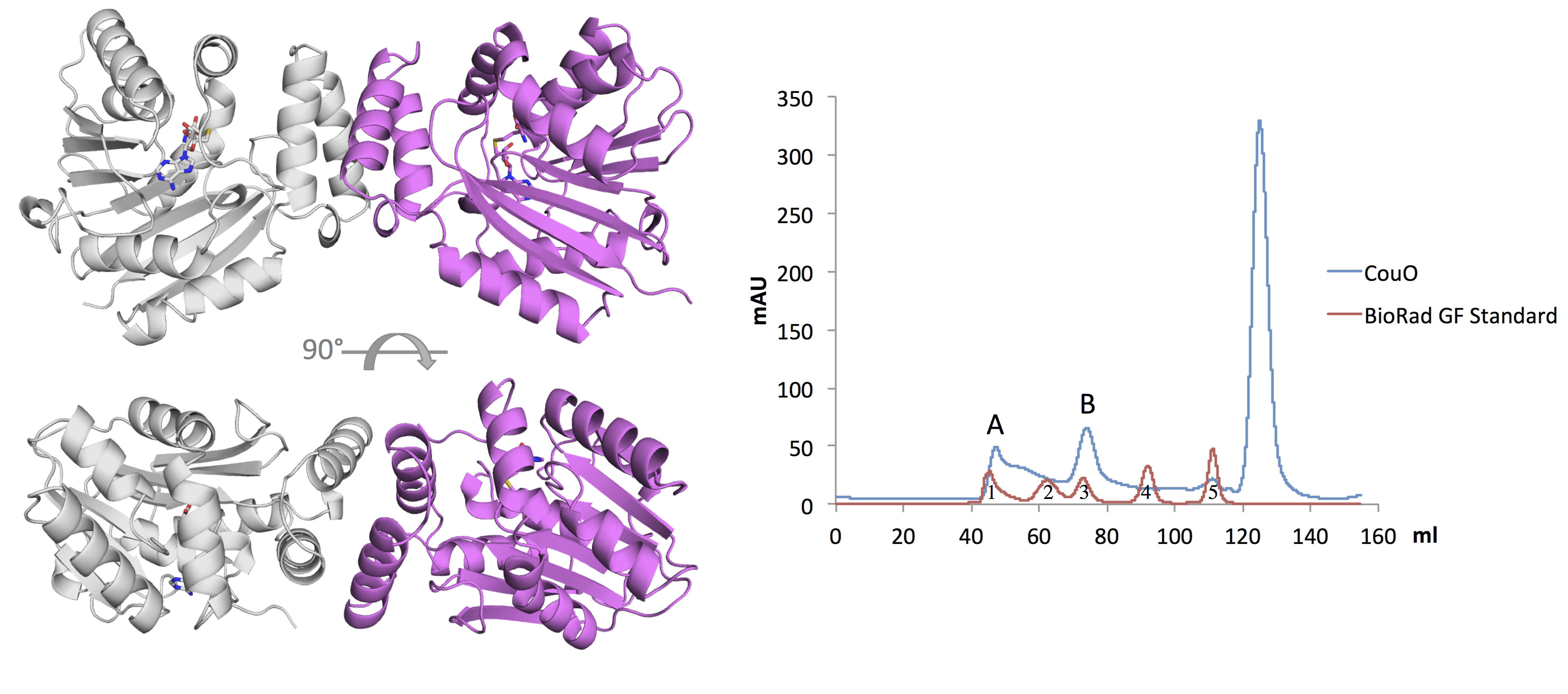

Supplement: S2 Fig — A) CouO dimer interface formed between the α-helices of the cap-domain that mainly involves hydrophobic interactions as identified by the PDBePISA server. The SAH cofactor is shown in sticks. B) The final step of CouO purification involved size exclusion chromatography (HiLoad 16/60 Superdex 200 pg column (GE Healthcare), 1 ml/min, Hepes pH 7, 100mM NaCl). Red trace: Bio-Rad gel filtration standard peaks: 1–670 kDa, thyroglobulin, 2–158 kDa, γ-globulin, 3–44k Da, ovalbumin, 4–17 kDa, myoglobin, 5–1.34 kDa, vitamin B12. In the blue trace, peak A corresponds to aggregates, whereas samples from peak B were used for crystallization. A least-squares fit (log(MW) vs. elution volume) yielded a molecular weight of 52.4 kDa for peak B. (TIF) [file pone.0171056.s002.tif]

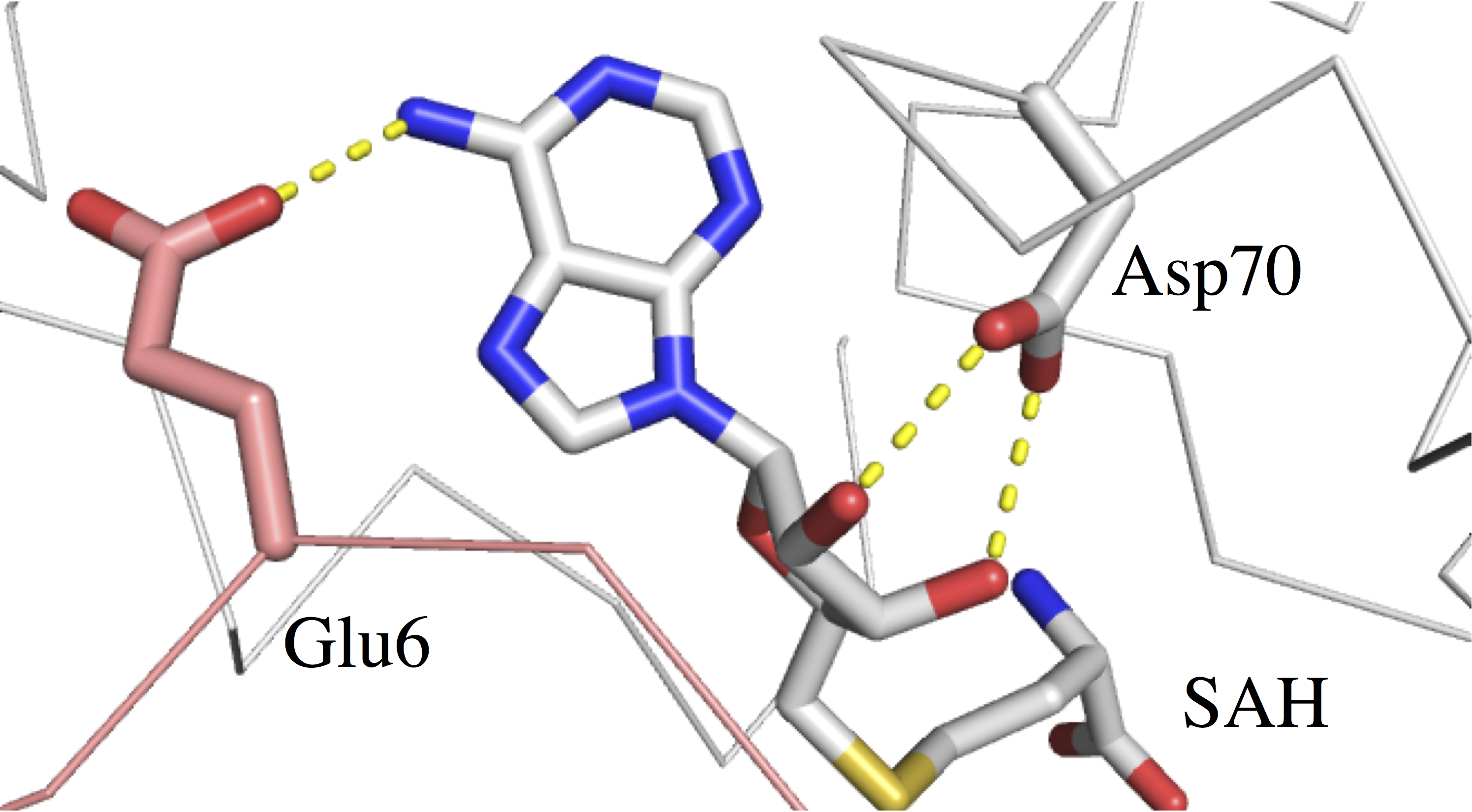

Supplement: S3 Fig — Amino acid residues present in the canonical motif, Asp70, and in the N-terminal tail, Glu6, are shown in a stick representation. The coloring scheme is the same as in Fig 1. Hydrogen bonding interactions are shown as yellow dashed lines. (TIF) [file pone.0171056.s003.tif]

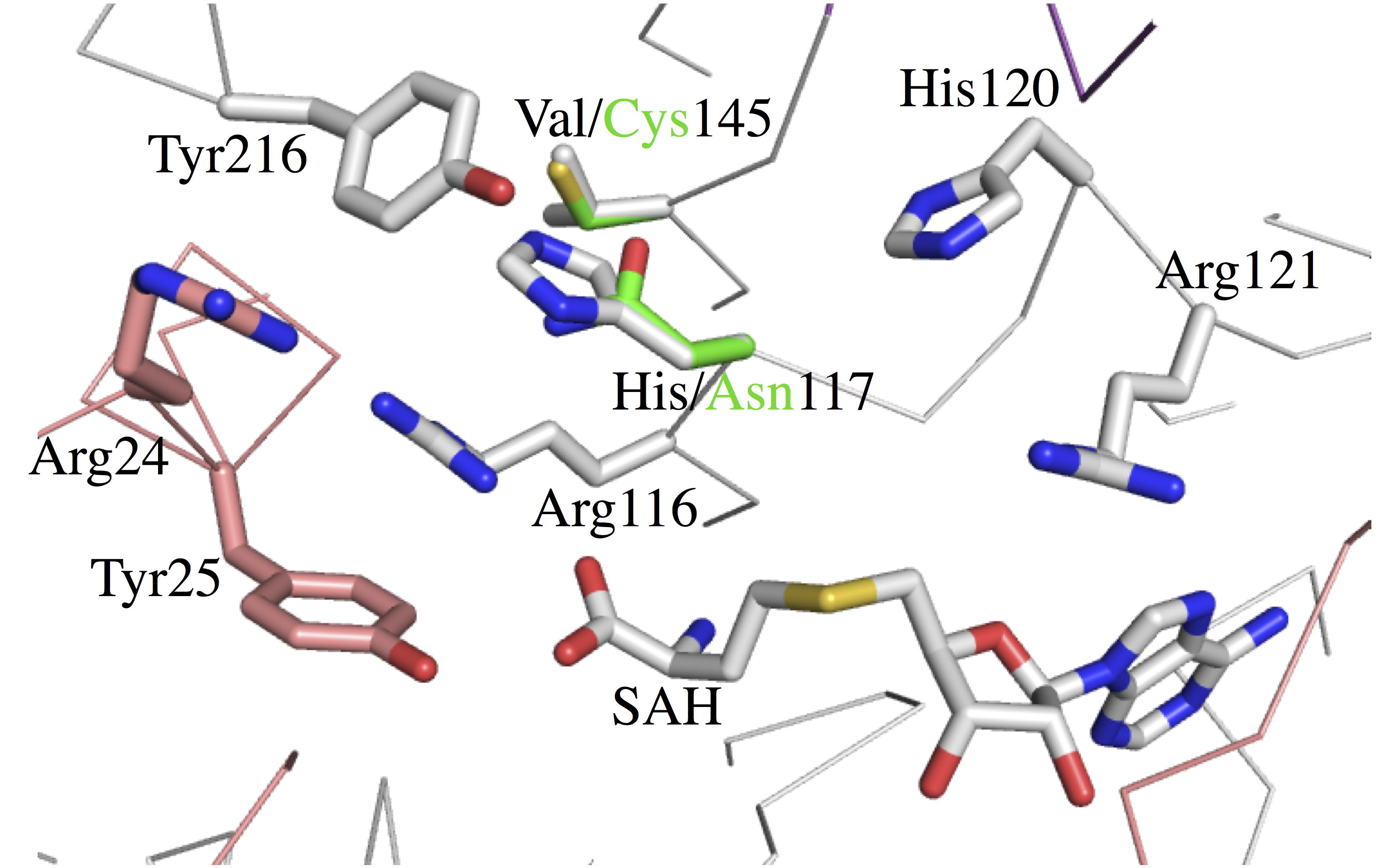

Supplement: S4 Fig — A homology model of NovO was generated using the CouO structure as a template (85% sequence identity, 90% similarity). Selected amino acid residues in and around the active site are shown in a stick representation. Residues 117 and 145 differ in two enzymes. The coloring scheme for CouO is the same as in Fig 1, residues differing in NovO are shown in green. (TIF) [file pone.0171056.s004.tif]

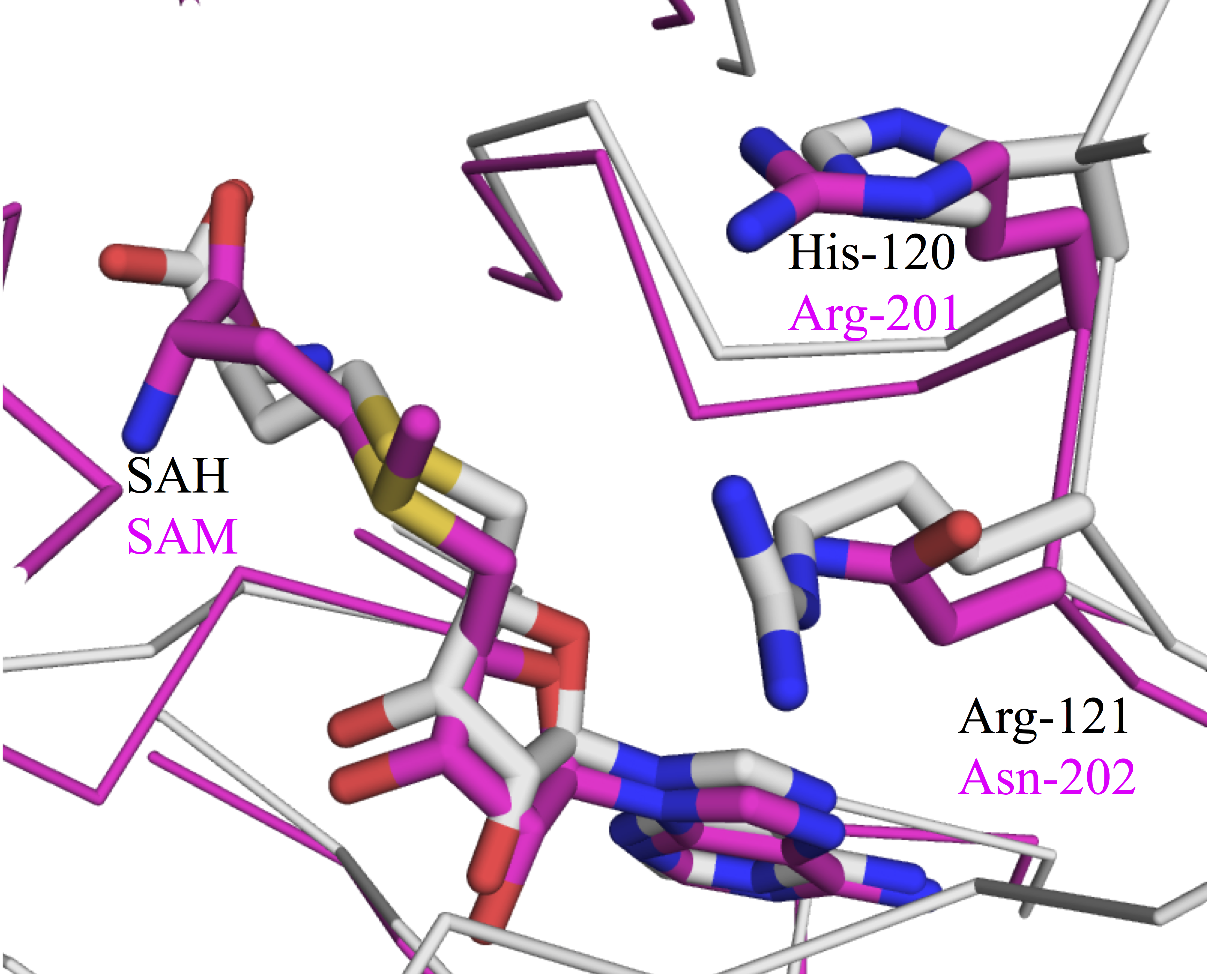

Supplement: S5 Fig — The residues suggested to be involved in catalysis, as well as the SAH/SAM cofactor are shown in sticks representations. (TIF) [file pone.0171056.s005.tif]

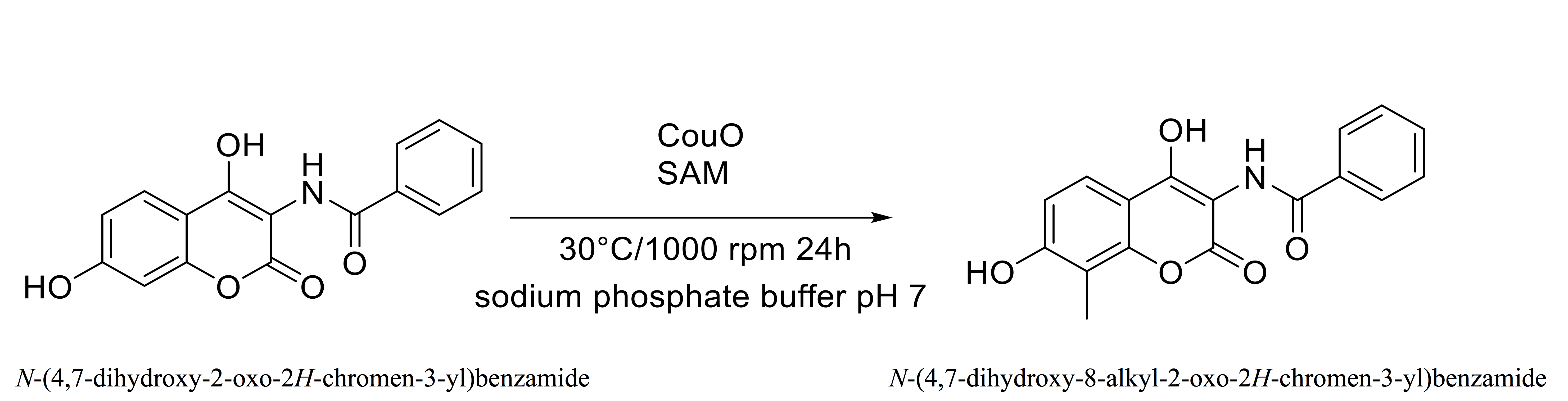

Supplement: S6 Fig — 0.5mM N-(4,7-dihydroxy-2-oxo-2H-chromen-3-yl)benzamide was used as substrate. (TIF) [file pone.0171056.s006.tif]

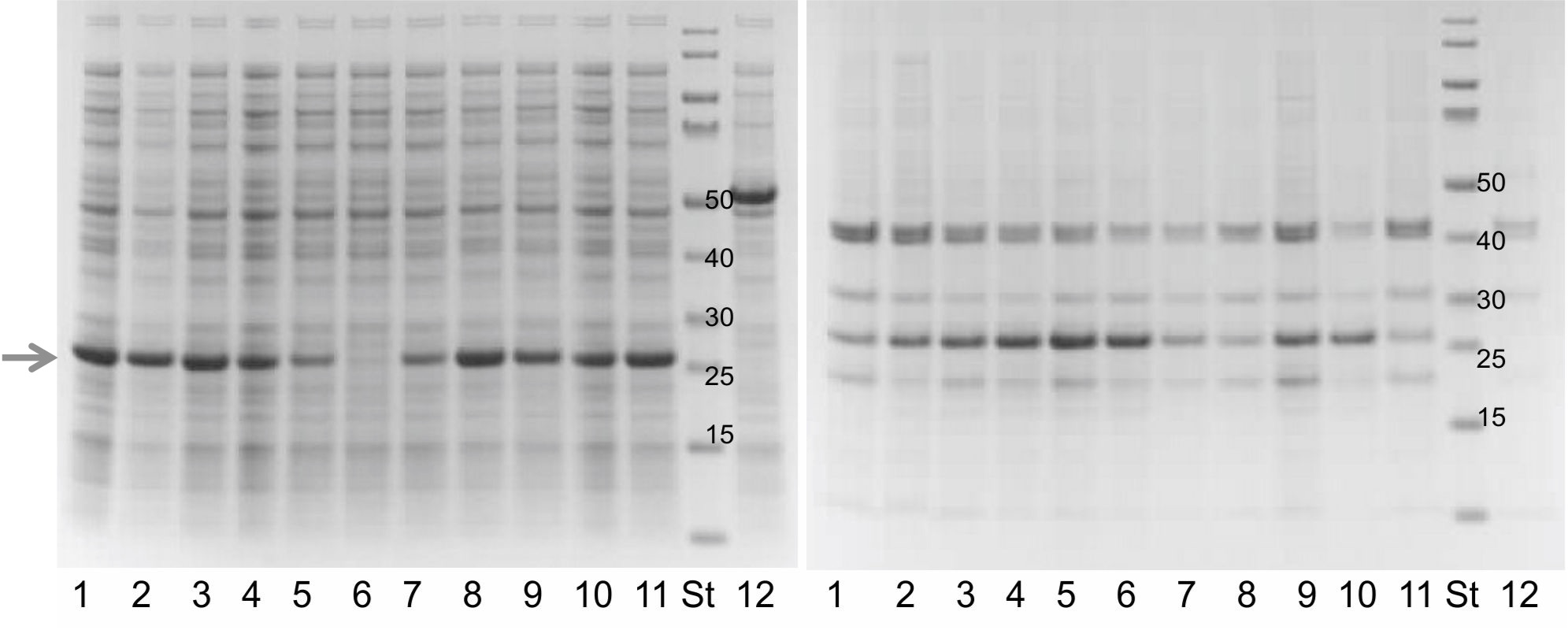

Supplement: S7 Fig — 1: H15A, 2: H15N, 3: R24A, 4: H117A, 5: H117S, 6: H120A, 7: H120N, 8: R121A, 9: R121L, 10: Y216F, 11: WT, 12: pMS470d8. St: Page Ruler Prestained Protein Ladder (Fermentas). The arrow indicates the location of the target protein (expected MW = 26kDa). (TIF) [file pone.0171056.s007.tif]
