# Supplementary material for: Crystal Structure and Catalytic Mechanism of CouO, a Versatile C-Methyltransferase from Streptomyces rishiriensis
Source: PLoS One. 2017 Feb 2;12(2):e0171056. doi: 10.1371/journal.pone.0171056 (PMC5289526; doi:10.1371/journal.pone.0171056)
Supplement: S1 File — (PDF containing 12 figures, Figures A to L in S1 File) (PDF) [file pone.0171056.s008.pdf]

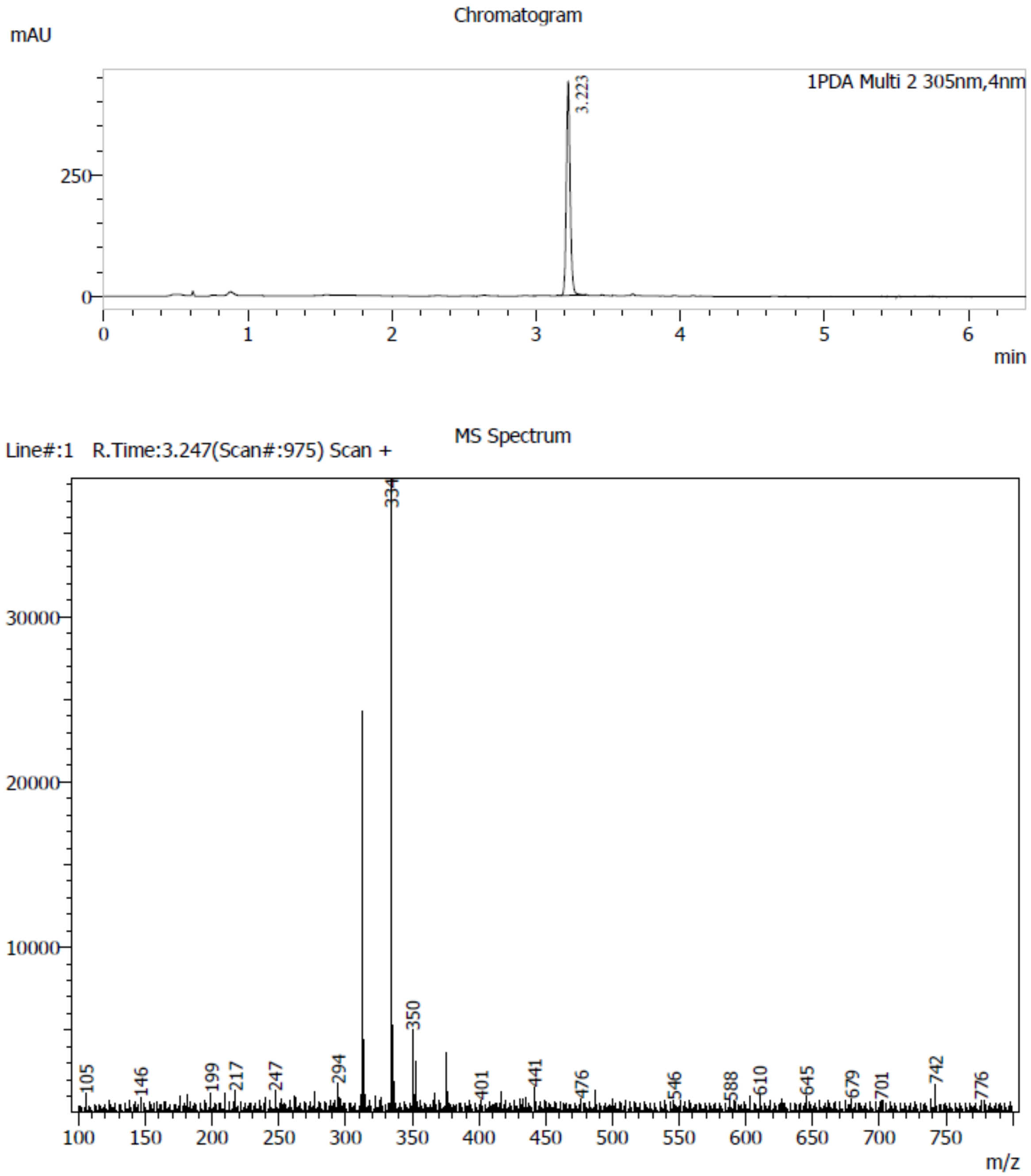

Figure A in S1 File. HPLC (UV) chromatogram and MS analysis of the conversion of *N*-(4,7-dihydroxy-2-oxo-2*H*-chromen-3-yl)benzamide catalyzed by the wild type enzyme. [M+H]+ = 312 m/z, [M+Na]+ = 334 m/z, [M+K]+ = 350 m/z

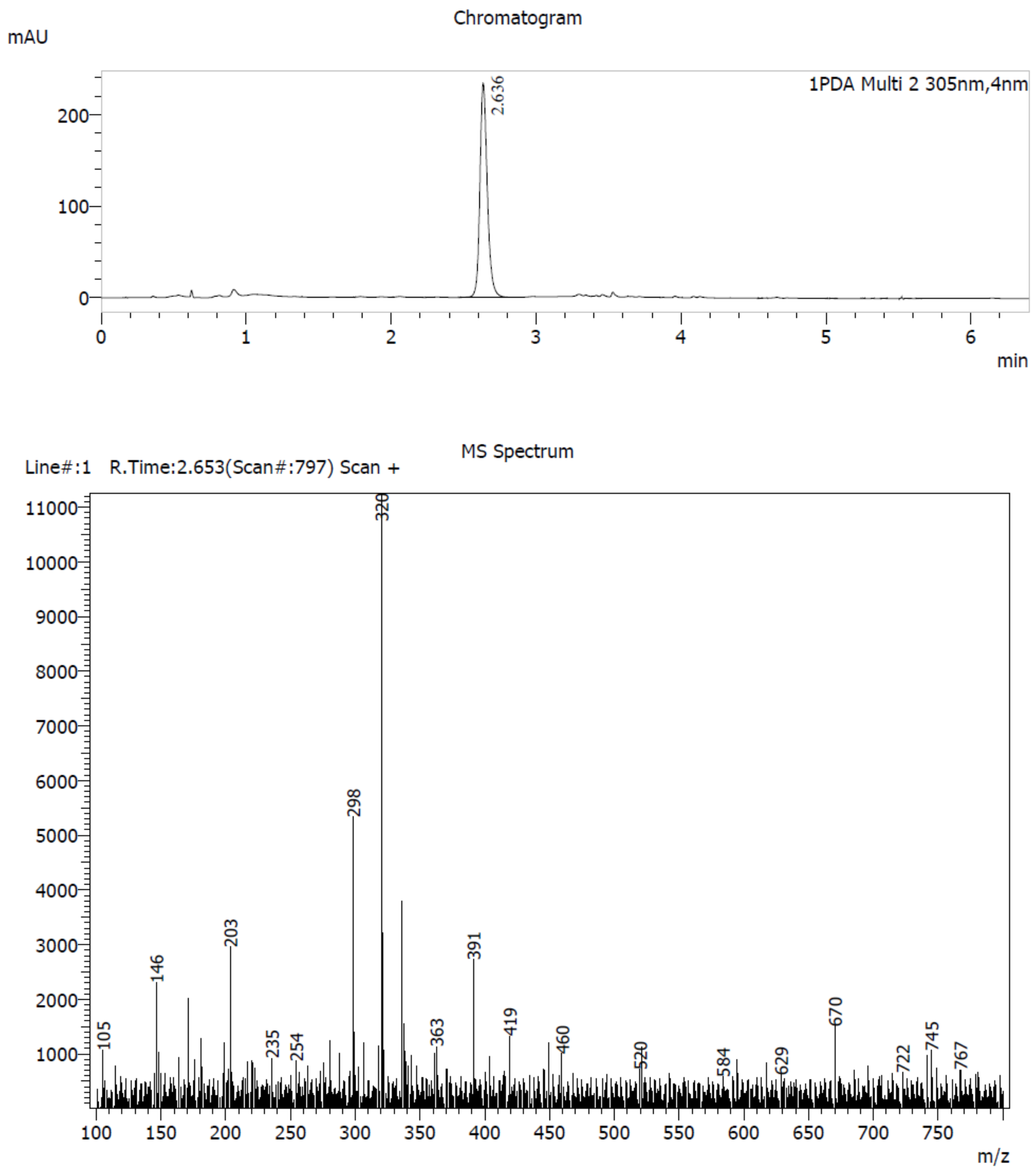

Figure B in S1 File. HPLC (UV) chromatogram and MS analysis of the blank reaction (50 mM sodium phosphate buffer pH 7). [M+H]+ = 298 m/z, [M+Na]+ = 320 m/z

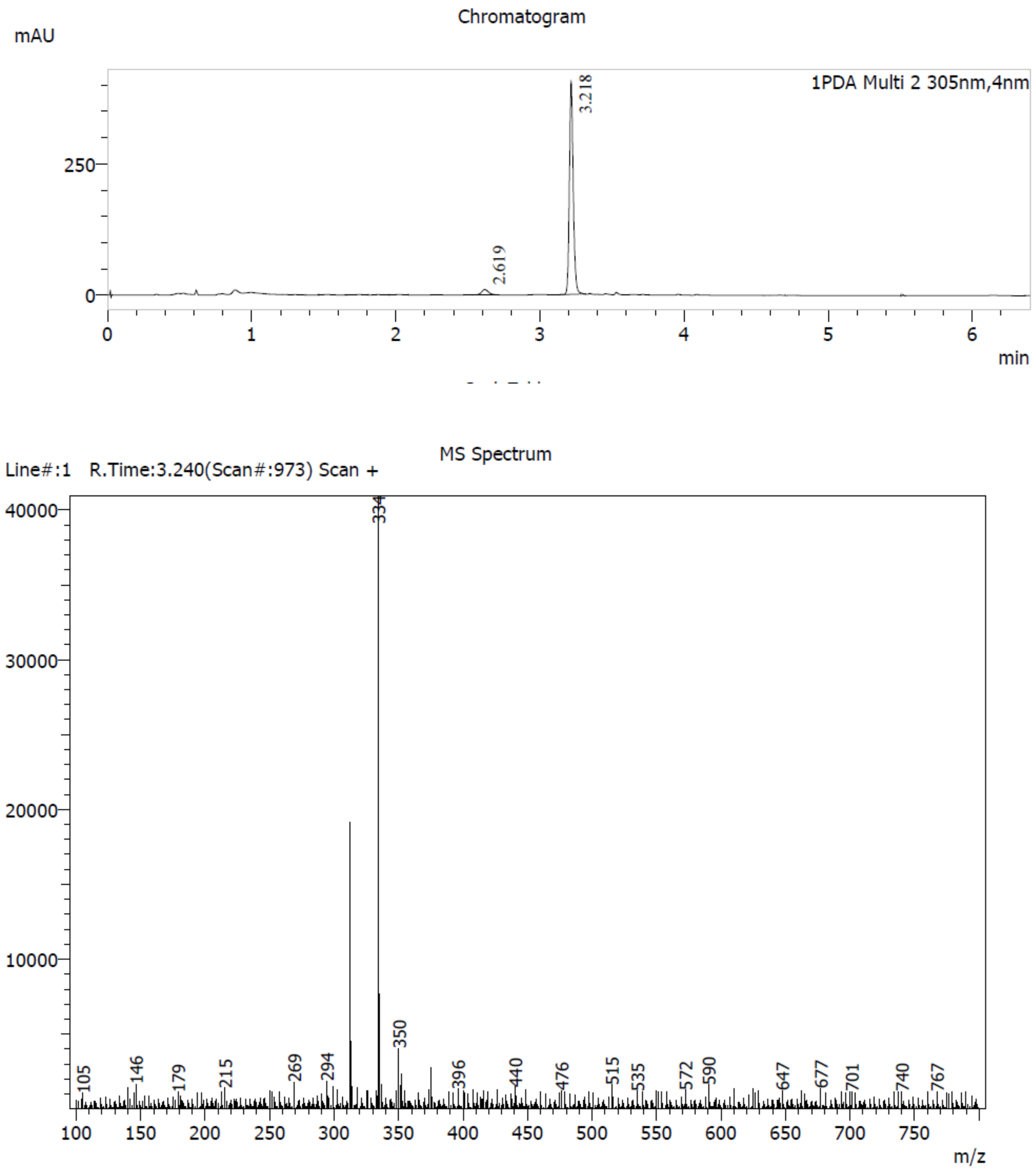

Figure C in S1 File. HPLC (UV) chromatogram and MS analysis of the conversion of *N*-(4,7-dihydroxy-2-oxo-2*H*-chromen-3-yl)benzamide catalyzed by the H15A variant.

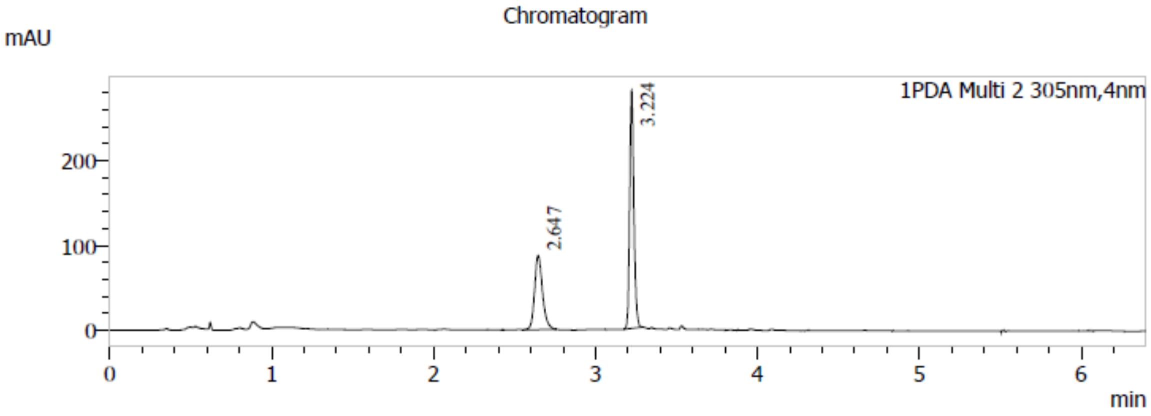

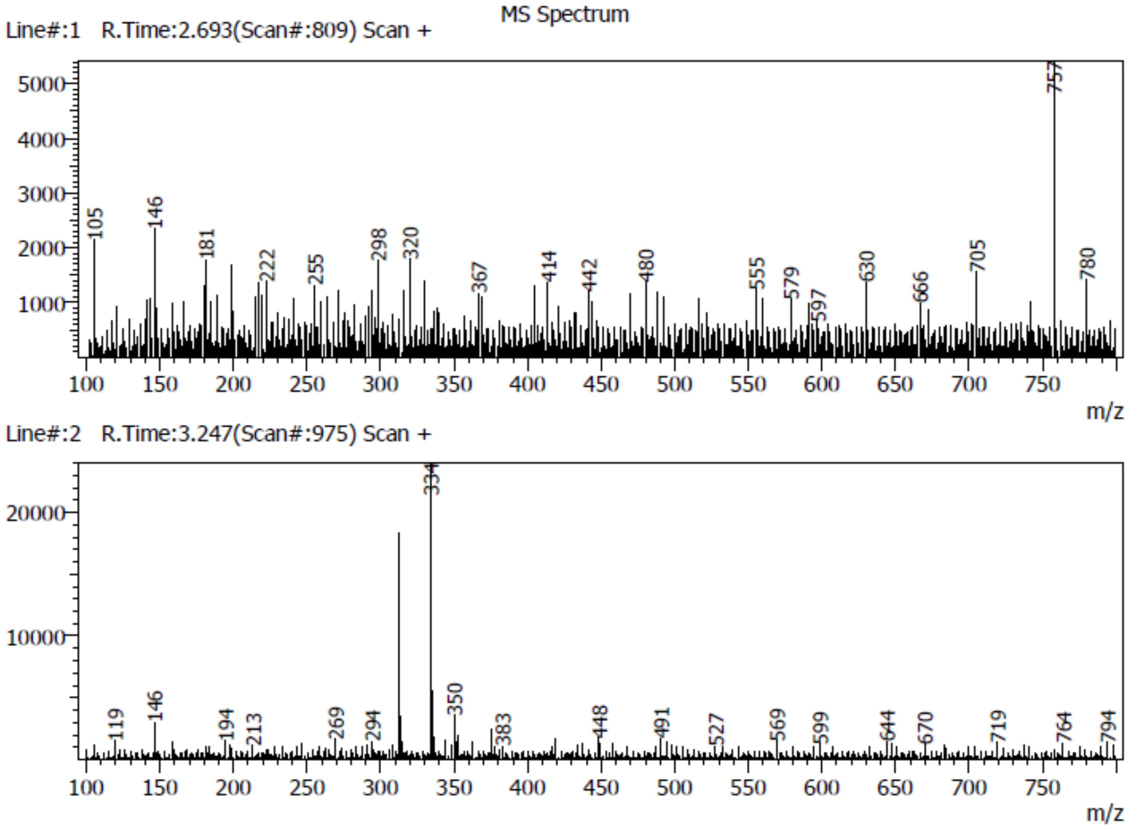

Figure D in S1 File. HPLC (UV) chromatogram and MS analysis of the conversion of *N*-(4,7-dihydroxy-2-oxo-2*H*-chromen-3-yl)benzamide catalyzed by the H15N variant.

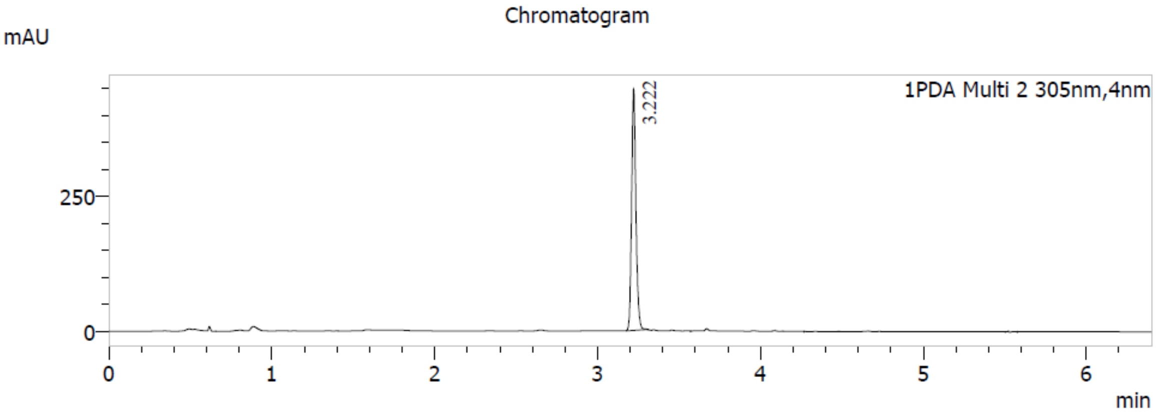

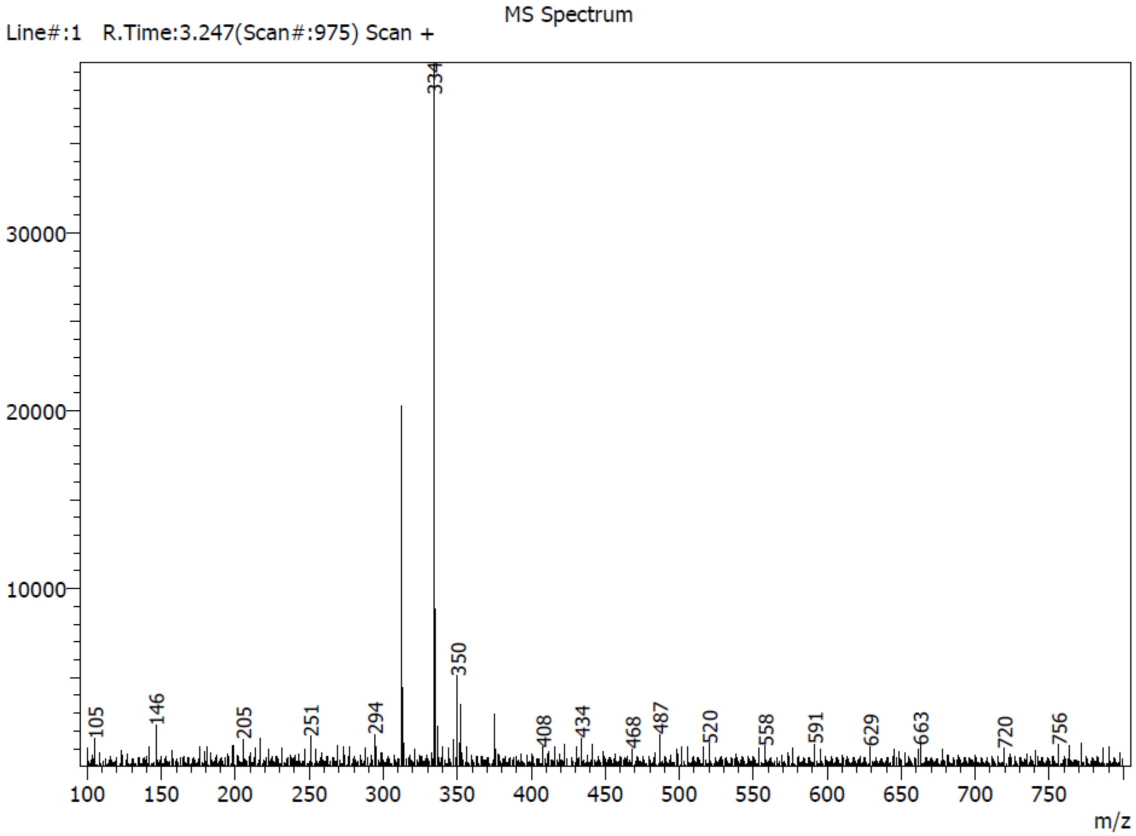

Figure E in S1 File. HPLC (MS) chromatogram and MS analysis of the conversion of *N*-(4,7-dihydroxy-2-oxo-2*H*-chromen-3-yl)benzamide catalyzed by the R24A variant.

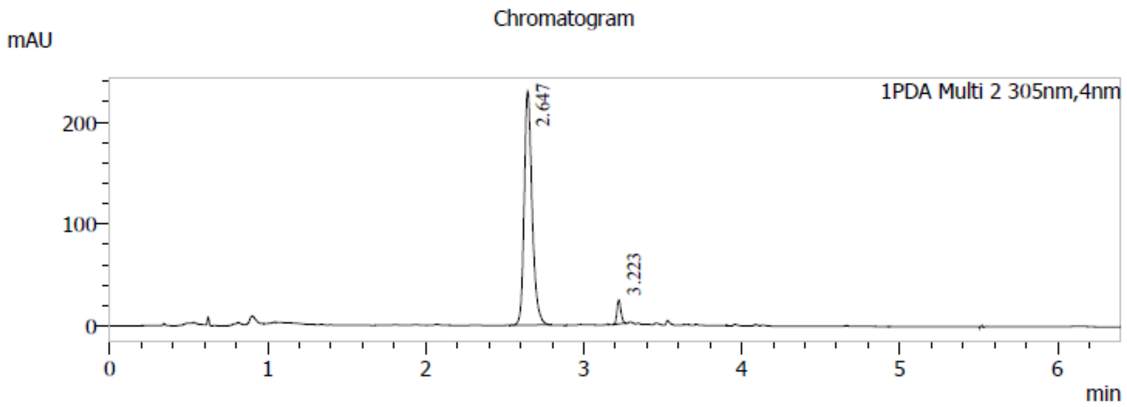

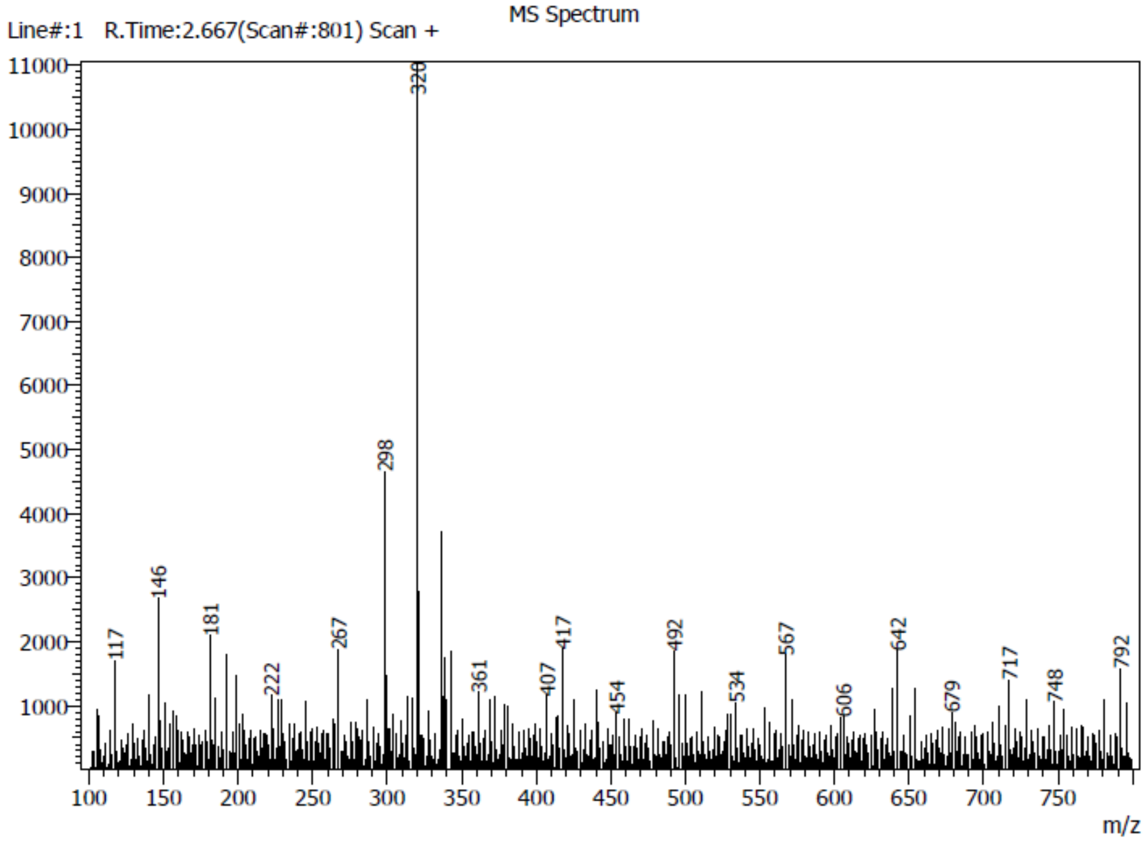

Figure F in S1 File. HPLC (UV) chromatogram and MS analysis of the conversion of *N*-(4,7-dihydroxy-2-oxo-2*H*-chromen-3-yl)benzamide catalyzed by the H117A variant.

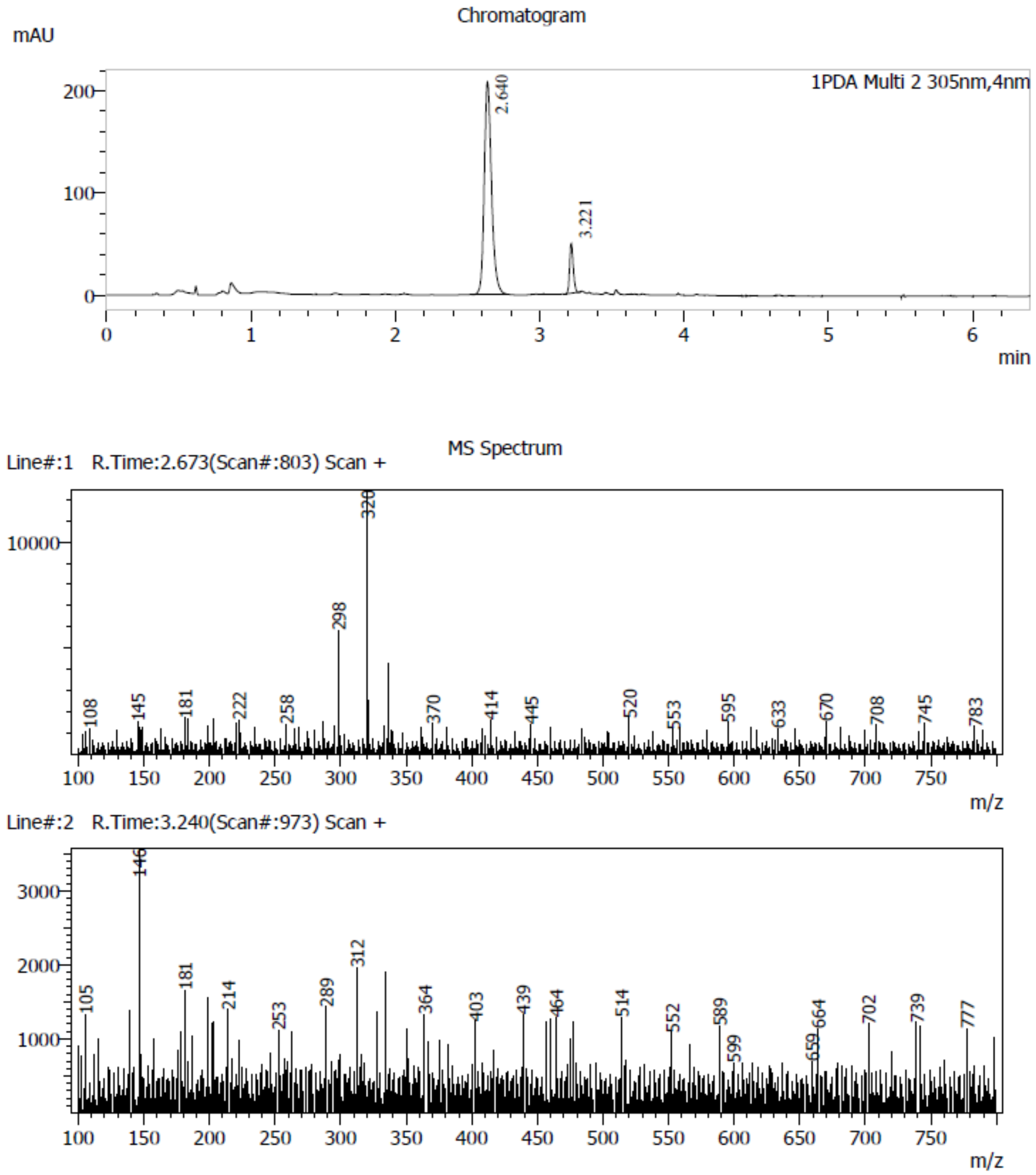

Figure G in S1 File. HPLC (MS) chromatogram and MS analysis of the conversion of *N*-(4,7-dihydroxy-2-oxo-2*H*-chromen-3-yl)benzamide catalyzed by the H117S variant.

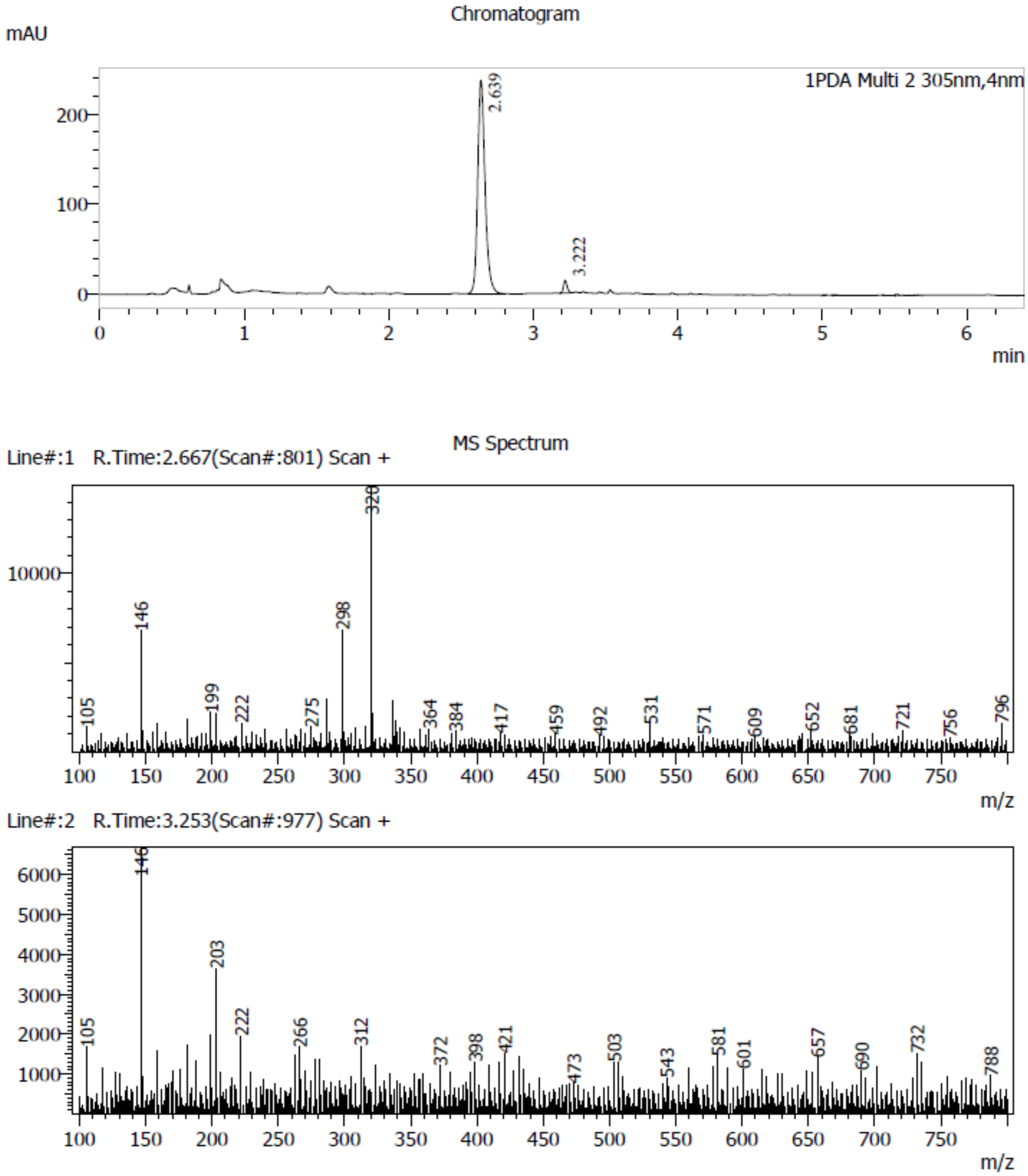

Figure H in S1 File. HPLC (UV) chromatogram and MS analysis of the conversion of *N*-(4,7-dihydroxy-2-oxo-2*H*-chromen-3-yl)benzamide catalyzed by the H120A variant.

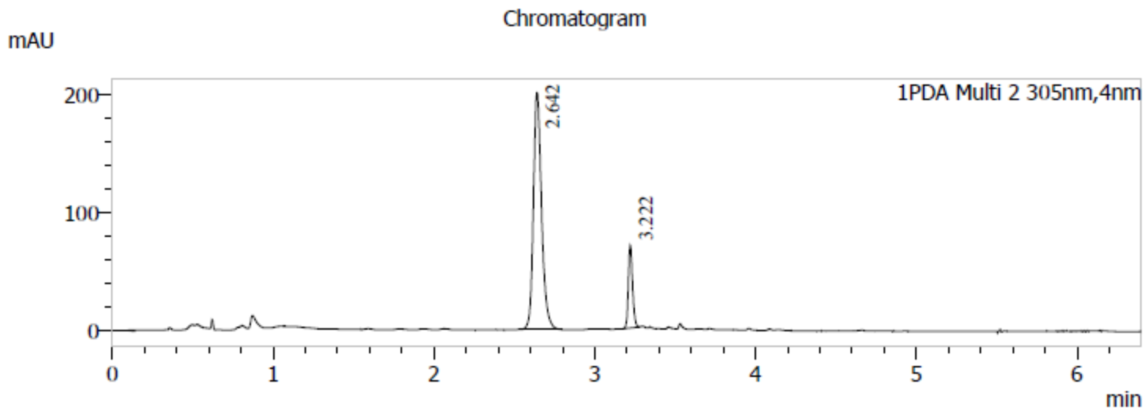

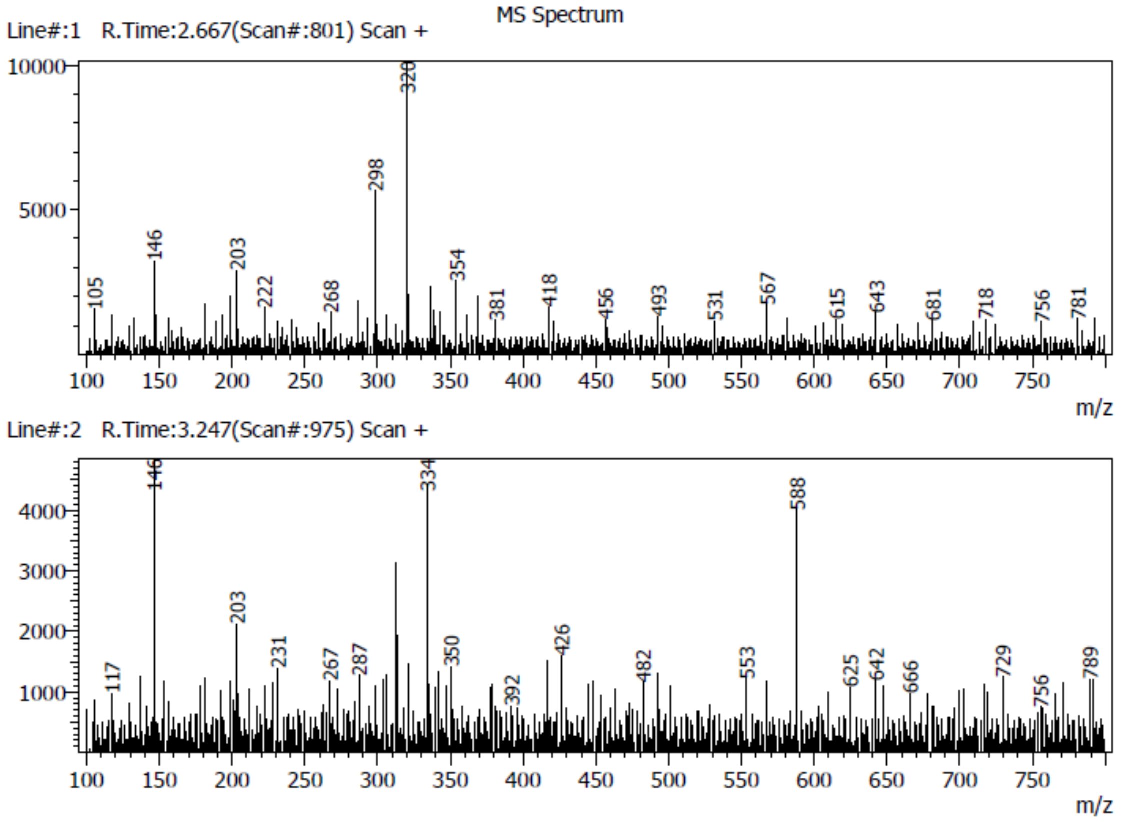

Figure I in S1 File. HPLC (UV) chromatogram and MS analysis of the conversion of *N*-(4,7-dihydroxy-2-oxo-2*H*-chromen-3-yl)benzamide catalyzed by the H120N variant.

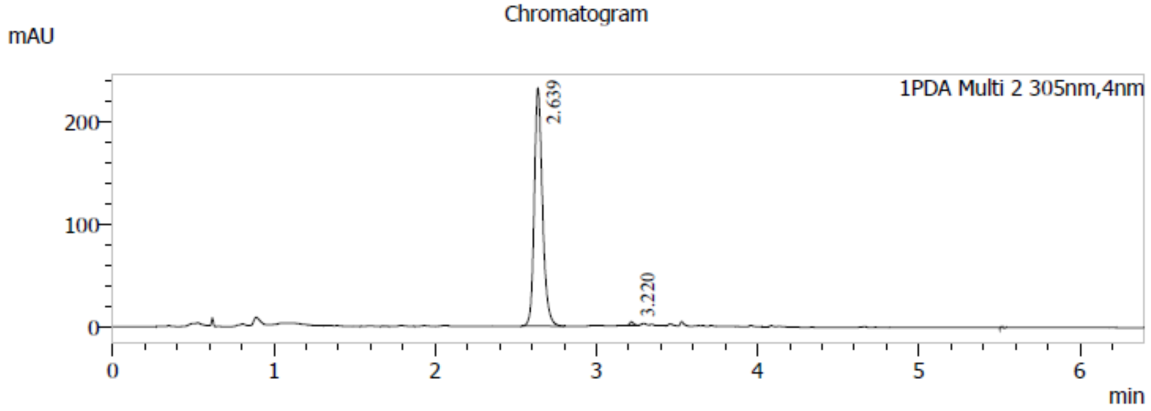

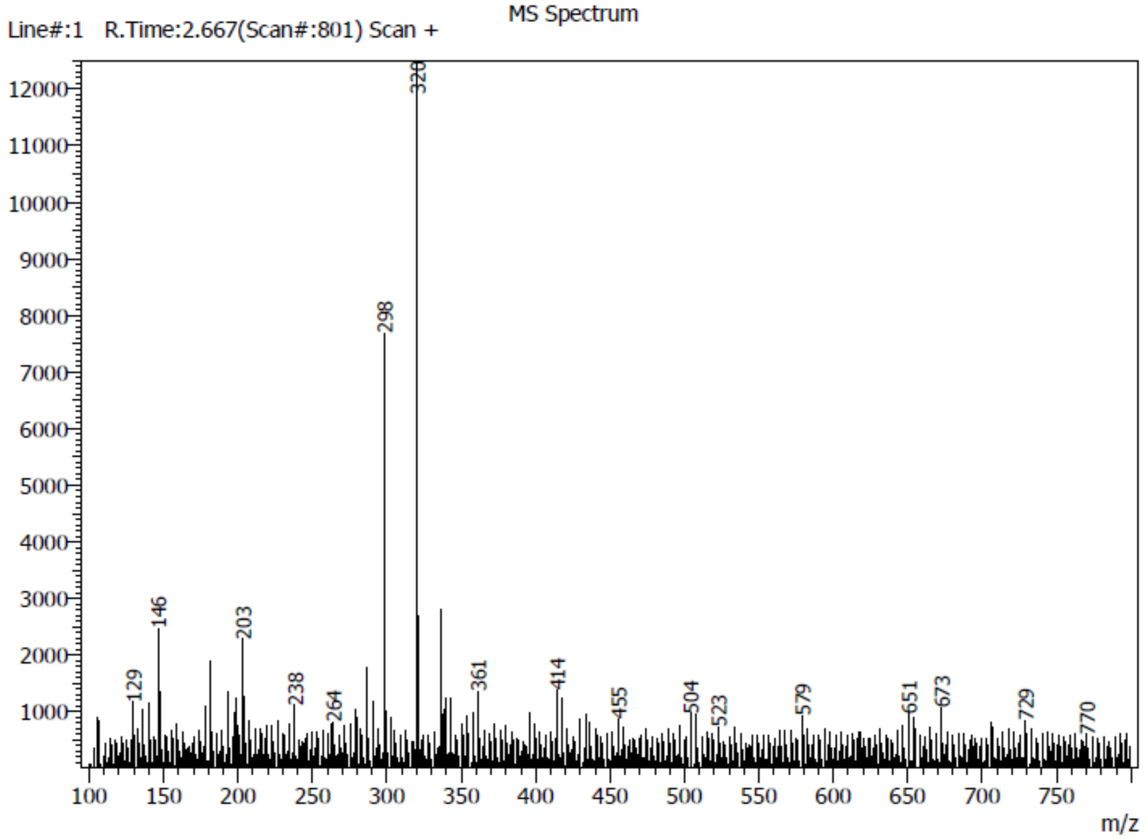

Figure J in S1 File. HPLC (UV) chromatogram and MS analysis of the conversion of *N*-(4,7-dihydroxy-2-oxo-2*H*-chromen-3-yl)benzamide catalyzed by the R121A variant.

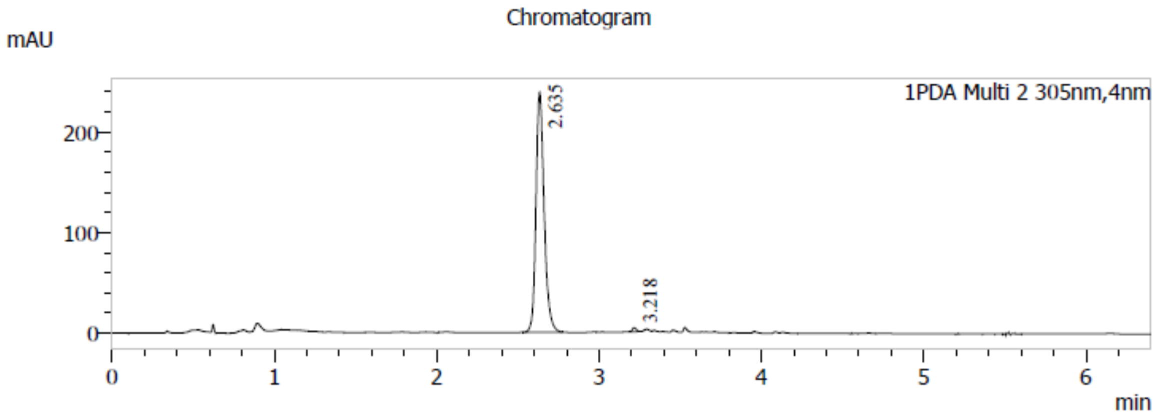

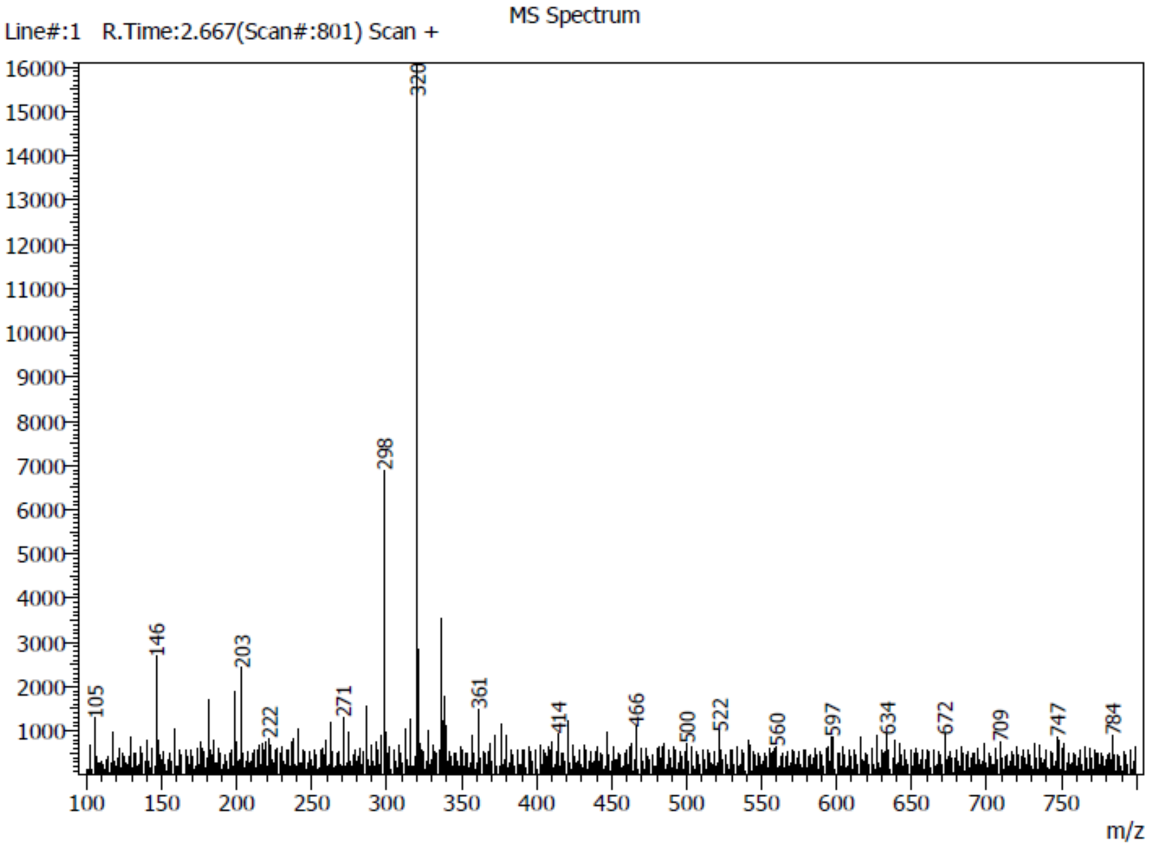

Figure K in S1 File. HPLC (UV) chromatogram and MS analysis of the conversion of *N*-(4,7-dihydroxy-2-oxo-2*H*-chromen-3-yl)benzamide catalyzed by the R121L variant.

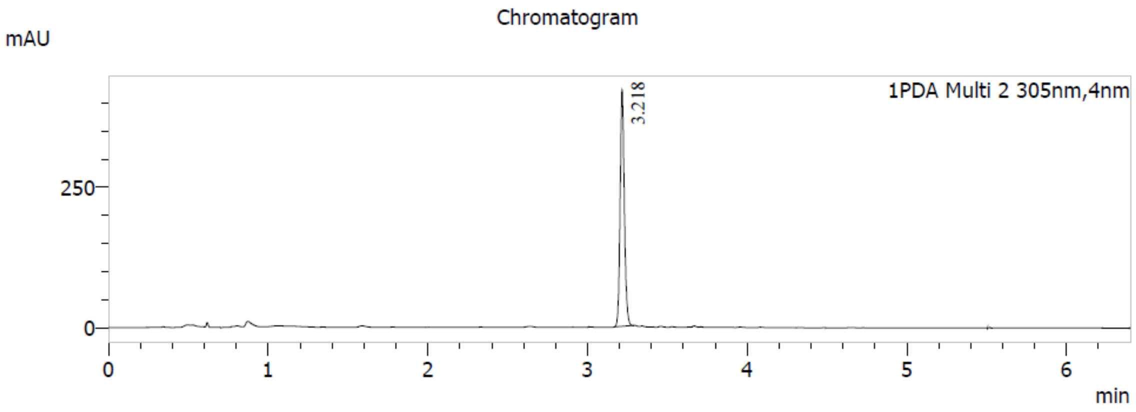

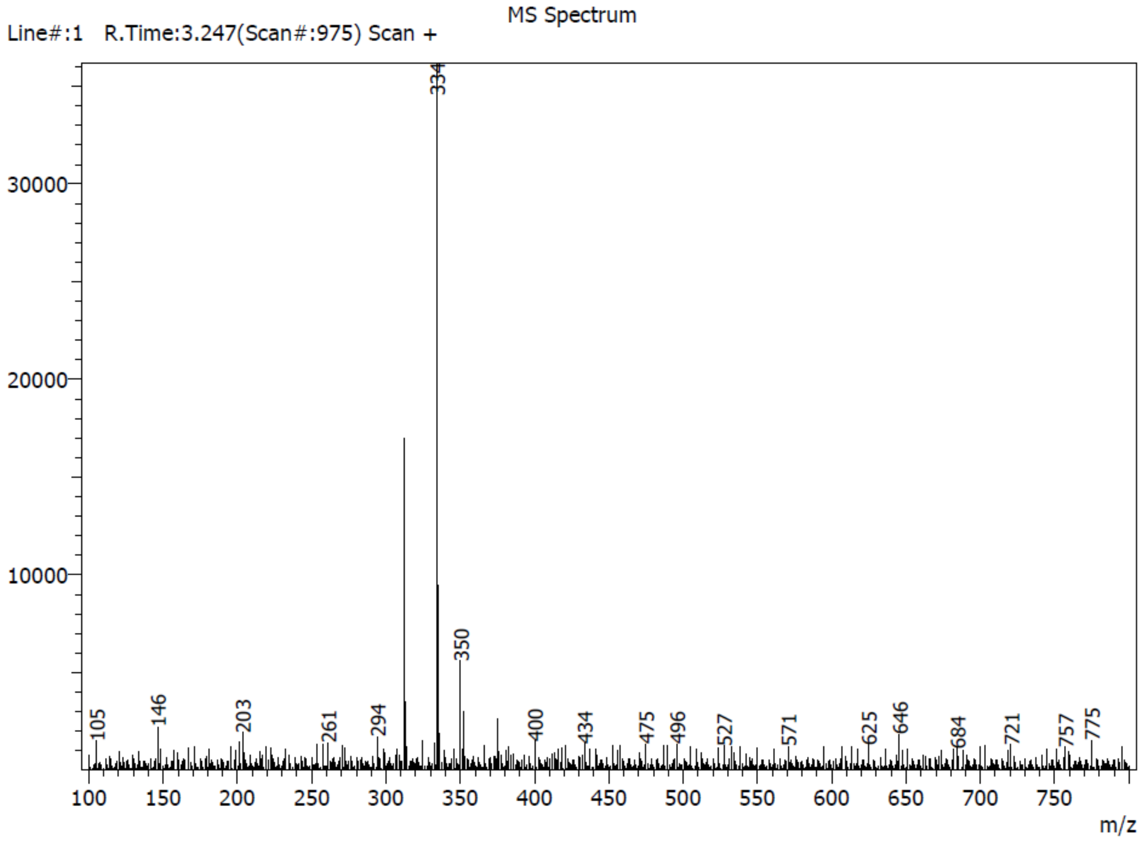

Figure L in S1 File. HPLC (UV) chromatogram and MS analysis of the conversion of *N*-(4,7-dihydroxy-2-oxo-2*H*-chromen-3-yl)benzamide catalyzed by the Y216F variant.
