# Supplementary material for: Crystal Structure and Catalytic Mechanism of CouO, a Versatile C-Methyltransferase from Streptomyces rishiriensis
Source: PLoS One. 2017 Feb 2;12(2):e0171056. doi: 10.1371/journal.pone.0171056 (PMC5289526; doi:10.1371/journal.pone.0171056)
Supplement: S1 Table — Sequences of restriction sites are underlined, start and stop codon are indicated with boxes. Bold red letters indicate the mutated bases. (PDF) [file pone.0171056.s009.pdf]

Primer Name (amino acid exchange)	Sequence
CouO(NdeI)_for	AATCACATATGAAGATTGAACCGATTACGGG
CouO(HindIII)_rev	AATCAAAGCTTTCAGGCAGCGGC
CouO-H15A_for	CGAAGCCTTT**GC**CCGGATGGG
CouO-H15A_rev	CCCATCCGG**GC**AAAGGCTTCG
CouO-H15N_for	CCGAAGCCTTT**A**ACCGGATGGG
CouO-H15N_rev	CCCATCCGGT**T**AAAGGCTTCGG
CouO-R24A_for	CCGGGCGTTCGAA**GC**GTACAACGAGTTC
CouO-R24A_rev	GAACTCGTTGTAC**GC**TTCGAACGCCCGG
CouO-H117A_for	GTGGTCTCGCGG**GC**CGCGTTCCACCG
CouO-H117A_rev	CGGTGGAACGCG**GC**CCGCGAGACCAC
CouO-H117S_for	GTGGTCTCGCGG**TC**CGCGTTCCACCG
CouO-H117S_rev	CGGTGGAACGCG**GA**CCGCGAGACCAC
CouO-H120A_for	GGCACGCGTTC**GC**CCGGCTCACCC
CouO-H120A_rev	GGGTGAGCCGG**GC**GAACGCGTGCC
CouO-H120N_for	GGCACGCGTTC**A**ACCGGCTCACC
CouO-H120N_rev	GGTGAGCCGGT**T**GAACGCGTGCC
CouO-R121A_for	CACGCGTTCCAC**GC**GCTCACCCGGC
CouO-R121A_rev	GCCGGGTGAGC**GC**GTGGAACGCGTG
CouO-R121L_for	CACGCGTTCCACC**T**GCTCACCCGGC
CouO-R121L_rev	GCCGGGTGAGC**A**GGTGGAACGCGTG
CouO-Y216F_for	GATGACCAGGGCT**T**CGGCGTTGCCAC
CouO-Y216F_rev	GTGGCAACGCCG**A**AGCCCTGGTCATC
CouO(pMS)_for	AATAATTTTGTTTAACTTTAAGAAGGAGATATACATATGAAGATTGAACCGATTACGGG
CouO(pMS)_rev	TGAAAATCTTCTCTCATCCGCCAAAACAGCCAAGCTTTCAGGCAGCGGCCC
