# Supplementary material for: Crystal Structure and Catalytic Mechanism of CouO, a Versatile C-Methyltransferase from Streptomyces rishiriensis
Source: PLoS One. 2017 Feb 2;12(2):e0171056. doi: 10.1371/journal.pone.0171056 (PMC5289526; doi:10.1371/journal.pone.0171056)
Supplement: S2 Table — (PDF) [file pone.0171056.s010.pdf]

CouO variant	% CouO in lysate	mg/mL total protein concentration	mg/mL Mtase
WT	28.4	8.86	2.52
H15A	29.4	7.54	2.22
H15N	28.6	9.13	2.61
R24A	28.6	10.77	3.08
H117A	20.3	13.80	2.80
H117S	14.3	5.32	0.76
H120A	5.5	6.50	0.36
H120N	15.8	8.45	1.34
R121A	33.5	8.49	2.84
R121L	28.9	7.00	2.02
Y216F	19.2	10.78	2.07
